# CatRange enables robust prediction of enzyme variant kinetic regimes

**DOI:** 10.1093/pnasnexus/pgag309

**Published:** 2026-09-16

**Authors:** Karuna Anna Sajeevan, Abraham Osinuga, Arunraj B, Sakib Ferdous, Nabia Shahreen, Mohammed Sakib Noor, Shashank Koneru, Laura Mariana Santos-Correa, Rahil Salehi, Niaz Bahar Chowdhury, Randy Aryee, Brisa Calderon-Lopez, Supantha Dey, Ankur Mali, Rajib Saha, Ratul Chowdhury

**Affiliations:** Department of Chemical and Biological Engineering, Iowa State University, Ames, IA 50011, USA; The Center for Biorenewable Chemicals, Iowa State University, Ames, IA 50011, USA; Ames National Laboratory, Ames, IA 50011, USA; Srinivasa Ramanujan Institute for Basic Sciences, Pi Hills, 8th Mile, Velloor, Pampady, Kottayam, Kerala 686501, India; Department of Chemical and Biomolecular Engineering, University of Nebraska-Lincoln, Lincoln, NE 68588, USA; Department of Chemical and Biological Engineering, Iowa State University, Ames, IA 50011, USA; Department of Chemical and Biological Engineering, Iowa State University, Ames, IA 50011, USA; Department of Chemical and Biomolecular Engineering, University of Nebraska-Lincoln, Lincoln, NE 68588, USA; Department of Chemical and Biological Engineering, Iowa State University, Ames, IA 50011, USA; Department of Chemical and Biological Engineering, Iowa State University, Ames, IA 50011, USA; Department of Chemical and Biological Engineering, Iowa State University, Ames, IA 50011, USA; Department of Chemical and Biological Engineering, Iowa State University, Ames, IA 50011, USA; Department of Chemical and Biomolecular Engineering, University of Nebraska-Lincoln, Lincoln, NE 68588, USA; Department of Chemical and Biological Engineering, Iowa State University, Ames, IA 50011, USA; The Center for Biorenewable Chemicals, Iowa State University, Ames, IA 50011, USA; Department of Chemical and Biological Engineering, Iowa State University, Ames, IA 50011, USA; Department of Chemical and Biological Engineering, Iowa State University, Ames, IA 50011, USA; Department of Computer Science and Engineering, University of South Florida, Tampa, FL 33620, USA; Department of Chemical and Biomolecular Engineering, University of Nebraska-Lincoln, Lincoln, NE 68588, USA; Department of Chemical and Biological Engineering, Iowa State University, Ames, IA 50011, USA; The Center for Biorenewable Chemicals, Iowa State University, Ames, IA 50011, USA; Ames National Laboratory, Ames, IA 50011, USA

**Keywords:** enzyme kinetics, bio-aware machine learning, enzyme engineering, biocatalysis, database curation

## Abstract

Predicting enzyme kinetics directly from sequence remains a central challenge in computational biology, particularly for mutations at catalytically essential residues. Existing models often default to wild-type predictions even when mutations cause substantial activity loss. This limits their utility for enzyme design and mechanistic inference. Here, we introduce CatRange, a machine learning framework trained on CatLog-27k, a human-in-the-loop, AI-agent curated dataset of 27,176 in vitro enzyme–substrate kinetic records. CatLog-27k was created through systematic audit and correction of BRENDA and SABIO-RK, with mutant entries manually reconciled against 2,158 source articles. CatRange reframes kinetic prediction as classification into log_10_-spaced bins for catalytic turnover (*k*_*cat*_) and substrate affinity (*K_M_*), rather than exact-value regression. This formulation reflects the order-of-magnitude scale at which enzyme kinetics are commonly interpreted. It reduces sensitivity to assay variability while preserving distinctions among functional catalytic and binding states. Using joint enzyme–substrate representations and gradient-boosted classifiers, CatRange predicts kinetic ranges for wild-type and mutant enzymes across held-out, out-of-distribution, and few-shot mutation settings. The model achieves robust order-of-magnitude recovery and class-balanced discrimination. Moreover, it captures mutation-induced transitions across kinetic regimes, including activity losses caused by perturbations of annotated catalytic residues. CatRange also identifies nonenzyme sequence inputs. Together, CatLog and CatRange provide an interpretable, mutation-sensitive framework for enzyme engineering and kinetic metabolic modeling.

Significance Statement Enzyme kinetic parameters determine how reactions proceed, yet measuring catalytic turnover (*k*_*cat*_) and substrate affinity (*K_M_*) remains slow, costly, and sensitive to assay conditions. Existing computational models are limited by noisy public databases and weak-to-no sensitivity to mutations at catalytic residues. CatRange addresses these challenges by combining CatLog-27k. CatLog is an audited-and-curated enzyme–substrate kinetics dataset by human-in-the-loop, agentic AI review of 2,158 articles, with guided catalytic-site variants generated by alanine substitution of active residues. Rather than predicting fragile point estimates, CatRange assigns kinetic parameters to order-of-magnitude regimes and evaluates predictions within a meaningful ±1-bin tolerance. This framework enables robust, interpretable, mutation-aware prediction of enzyme kinetic behavior for enzyme engineering and kinetic metabolic modeling.

## Introduction

Precise characterization of enzyme kinetics is foundational for synthetic biology, systems biology, biomanufacturing, and disease-relevant metabolism, where catalytic turnover and substrate-dependent activity shape pathway performance and cellular phenotypes (1, 2). Predicting enzyme kinetic parameters from sequence and substrate information could accelerate metabolic reconstruction in nonmodel organisms, guide enzyme engineering for specific substrates, and reduce reliance on costly experimental screening. Such predictive capability is particularly valuable when sequence variation alters enzyme function, whether through engineered mutations, naturally occurring polymorphisms, or disease-associated variants. Because substitutions at functionally important residues can shift catalytic behavior by orders of magnitude, computational models for enzyme kinetics must be both accurate on average and sensitive to mutation-associated changes in catalytic and apparent-affinity regimes (3, 4). The difficulty of capturing these precise shifts is further amplified by the gap between in vitro kinetic measurements and the dynamic biochemical environments of cells, where metabolite availability, regulation, and cellular context constrain the effective activity realized in vivo.

Machine learning has enabled substantial advances in enzyme kinetics prediction. DLKcat, TurNuP, UniKP, CatPred, and EITLEM-Kinetics have shown that protein representations, molecular fingerprints, graph-based encodings, environmental descriptors, and transfer-learning strategies can improve prediction of enzyme–substrate kinetic parameters across diverse datasets (5–9). These models have helped establish enzyme kinetics as a tractable data-driven prediction problem, with recent approaches reporting substantial fractions of predictions within one order of magnitude of experimental values. CatPred, for example, leverages neural networks trained on SABIO-RK and BRENDA (10, 11), reporting ∼76% of *k*_*cat*_ predictions and 84% of *K_M_* predictions within one order of magnitude (9). Despite these advances, reliable kinetic prediction remains constrained by three linked challenges. *First,* measurements are sparse, unevenly distributed across enzyme families, and sensitive to assay conditions—including pH, temperature, buffer composition, co-substrate availability, enzyme source, and construct design—making pooled numerical targets intrinsically noisy. *Second,* public kinetic resources contain inconsistencies in literature provenance and duplicate handling, necessitating a data audit (between source articles and public databases) before any downstream predictive model building effort. This involves rigorous, falsifiable, and reproducible protocols to reconcile and standardize training data sources into a unified corpus to enable fair model comparison. *Third,* mutation-sensitive prediction remains particularly difficult because catalytic-site substitutions can preserve near-complete global sequence identity while disrupting acid–base chemistry, nucleophilic attack, metal coordination, substrate positioning, or transition-state stabilization. Consequently, when mutation-specific data are sparse, models optimized for continuous regression or aggregate performance frequently fail to resolve functional departures from wild-type (WT) behavior in highly similar variants (12).

For enzyme engineering and functional variant interpretation, the field requires a framework that moves beyond continuous point-estimation to explicitly capture functionally consequential shifts. We therefore introduce CatRange, a classification-based framework that predicts enzyme kinetic regimes using paired enzyme sequence (13) and substrate (14) embeddings. CatRange is trained on CatLog-27k—a rigorously curated collection of 27,176 kinetic records reconciled against 2,158 primary source articles, which yields 24,517 unique median-pooled sequence–substrate entries, and augmented with 5,278 synthetic catalytic-site alanine substitutions calibrated against literature-derived in vitro experimental anchors. By reformulating $k_{cat}$ and $K_{M}$ prediction as classification into log_10_-spaced kinetic regimes (discrete numerical bins), CatRange mitigates the instability of nominal pointwise agreement across heterogeneous assays. Instead, it evaluates whether an enzyme–substrate pair occupies the correct order-of-magnitude range using exact-bin discrimination and adjacent-bin *e-accuracy*. Evaluated across stringent homology-stratified splits, withheld catalytic-residue anchors, and an external alkaline phosphatase (PafA) mutational landscape, CatRange demonstrates robust generalization and sensitivity to functionally consequential mutations. Ultimately, these regime-level estimates enable actionable kinetic prioritization in early-stage enzyme engineering and provide bounded kinetic parameters for metabolic modeling (15), that better accommodate the inherent uncertainty between in vitro measurements and effective cellular rates (16–18).

## Results

### CatLog-27k provides a curated enzyme–substrate kinetic landscape

CatLog-27k comprises 27,176 curated enzyme–substrate kinetic records assembled from SABIO-RK (11) and BRENDA (10) (Fig. 1A). Records were reconciled against UniProt (19), PubChem (20), and primary literature to resolve enzyme identity, sequence context, substrate assignment, mutation status, and reported catalytic turnover (*k*_*cat*_) and Michaelis constant (*K_M_*) values. Manual review of 2,158 source articles resolved 1,804 inconsistencies in kinetic values, enzyme sequences, substrate identities, or annotations (Table S1), highlighting the extent of reconciliation required before model development. Multisubstrate reactions were retained as substrate-specific records, consistent with the substrate-conditioned form in which kinetic parameters are commonly reported (21). An agent-assisted curation framework was developed in parallel to standardize these checks for future updates (see SI Appendix, Section S1.5).

**Figure 1:**
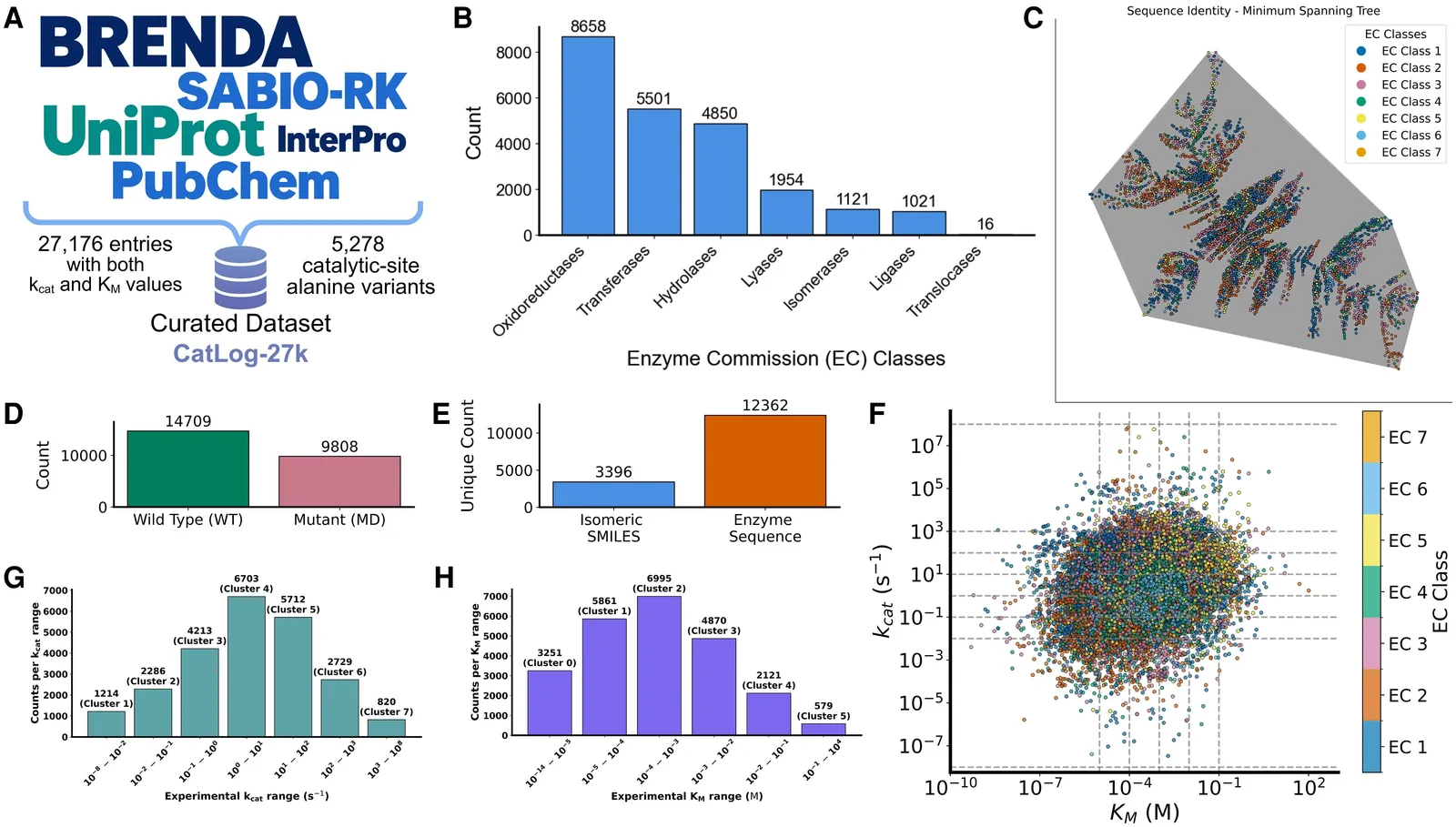
Construction and kinetic landscape of the CatLog-27k dataset. A) Integration of public enzyme-kinetics databases and sequence/substrate annotation resources yields a curated dataset of 27,176 enzyme–substrate entries with paired experimental *k*_*cat*_ and *K_M_* values. The dataset is supplemented by 5,278 synthetic catalytic-site alanine variants to anchor mutation-aware supervision. B) Distribution of the curated entries across the seven major EC classes. C) Minimum spanning tree illustrating sequence-identity relationships across CatLog-27k, colored by EC class. D) Distribution of experimentally validated WT and mutant (MD) entries following duplicate consolidation and median pooling. E) Absolute counts of unique substrate representations (isomeric SMILES) and enzyme sequences comprising the final dataset. F) Bivariate distribution of experimental *k*_*cat*_ and *K_M_* values, highlighting the broad dynamic range of the kinetic landscape across all EC classes. G and H) Order-of-magnitude, log-scaled kinetic bins for *k*_*cat*_ (G) and *K_M_* (H), which define the discrete multiclass prediction targets for the CatRange architecture.

CatLog-27k spans substantial enzymatic, chemical, and taxonomic diversity, including 3,396 unique isomeric SMILES, 12,362 enzyme sequences, and enzymes from 1,499 organisms (Fig. 1E). All major Enzyme Commission (EC) classes are represented, with oxidoreductases, transferases, and hydrolases contributing the largest fractions (Fig. 1B). A minimum spanning tree representation derived from pairwise sequence-identity network revealed heterogeneous local sequence relationships across CatLog-27k, with extensive intermixing of primary EC classes and localized class enrichment along some branches (Fig. 1C; SI Appendix, Section S1.4). Importantly, repeated measurements for identical sequence–substrate pairs remained highly variable even after curation, with extreme cases spanning several orders of magnitude (Fig. S2). This residual heterogeneity motivated median aggregation rather than selection of extreme reported values for model development.

After exclusion of sequences longer than 1,024 residues and consolidation of identical sequence–isomeric-SMILES pairs, median pooling yielded 24,517 unique sequence–substrate entries, comprising 14,709 WT and 9,808 mutant entries (Fig. 1D; SI Appendix, Section S1.3). This experimentally grounded landscape was supplemented with 5,278 synthetic catalytic-site alanine substitutions to introduce mutation-aware training instances (Fig. 1A), creating a training corpus that preserves measured kinetic diversity while introducing mutation-aware examples of catalytic perturbation. Crucially, the fully public CatLog-27k release preserves the prepooled individual measurements, literature provenance, and available assay metadata alongside the final model-ready representation, providing a transparent, interactive resource for the community and making the transformations used for CatRange development explicit.

### Log-scaled kinetic regimes capture functionally interpretable variation

Enzyme kinetic parameters vary over orders of magnitude, making exact point prediction sensitive to assay conditions, reporting differences, and residual experimental heterogeneity even after curation (Fig. S2). We therefore reformulated catalytic turnover (*k*_*cat*_) and Michaelis constant (*K_M_*) as log_10_-spaced kinetic ranges at order-of-magnitude resolution rather than continuous point targets (Fig. 1F–H), with each predefined numerical range treated as a discrete prediction bin (see SI Appendix, Section S2.1). This representation preserves order-of-magnitude differences while reducing reliance on nominal numerical agreement across heterogeneous experimental conditions. Edge classes were retained for extreme values so that rare kinetic measurements remained represented rather than being compressed into the central range.

To evaluate predictions at this resolution, we defined *e-accuracy* as the fraction of predictions assigned either to the correct experimental bin or to an immediately adjacent bin. While exact-bin accuracy measures strict multiclass discrimination, *e-accuracy* quantifies agreement within a ±1-bin tolerance. This distinction is critical because residual variability among repeated measurements can cross log-scale boundaries, with reported values for some identical enzyme–substrate pairs spanning up to 6–8 orders of magnitude across independent studies (Fig. S2). Adjacent-bin agreement therefore provides a tolerance-aware metric of whether the prediction recovers the experimental order of magnitude without enforcing an unrealistic exact-recovery standard. CatRange is consequently evaluated as a kinetic-regime (order-of-magnitude range) classifier for functional inference, reporting exact-bin and adjacent-bin performance separately rather than interpreting results as nominal point-estimation accuracy.

### Mechanistically informed alanine variants anchor mutation sensitivity

Catalytic-site mutations are sparsely represented in public enzyme-kinetics databases, yet they provide a stringent test of whether a model recognizes residues that directly shape enzymatic function. To introduce such perturbations into CatRange training, we supplemented the experimentally curated CatLog-27k landscape with 5,278 mechanistically guided catalytic-site alanine variants generated by substituting UniProt-annotated catalytic residues with alanine (Fig. 1A; SI Appendix, Section S2.4). Alanine substitution removes side-chain chemistry that may participate in acid–base catalysis, nucleophilic attack, metal coordination, substrate positioning, or transition-state stabilization, while preserving a sequence background that maintains near-perfect global sequence identity to the cognate wild type (WT). These synthetic alanine variants (MD_Ala_) therefore create difficult, sequence-proximal examples in which small, localized changes can produce large kinetic consequences.

Because catalytic-site alanine substitution does not uniformly abolish activity across all enzymes, residues, and substrates, we explicitly avoided imposing a blanket zero-activity label. Instead, the mutation-effect labels for these synthetic variants were calibrated by a meta-learner trained on 22 literature-derived in vitro WT–MD_Ala_ anchor pairs (Table S3; see SI Appendix, Sections S2.2–S2.4 for details on catalytic-site alanine calibration). To ensure leakage-aware evaluation, these experimental anchors pairs (WT–MD_Ala_) were constrained to ≤60% sequence identity relative to the CatRange training set. They were strictly withheld from classifier fitting and hyperparameter optimization, serving solely upstream to calibrate the synthetic-labeling policy. These anchors captured a realistic range of mutation-associated kinetic responses: some alanine substitutions produced large decreases in *k*_*cat*_, others primarily altered *K_M_*, and some remained within the same kinetic regime. This heterogeneity is biologically informative, as catalytic residues contribute differently to chemical turnover, substrate positioning, binding-site organization, and transition-state stabilization. The calibrated synthetic variants therefore served as hard counterexamples rather than simple inactive labels, forcing CatRange to distinguish localized catalytic relevance from global sequence identity. This design supports the central objective of mutation-aware kinetic prediction: namely, detecting functionally consequential shifts across kinetic regimes, rather than defaulting to WT-like predictions for highly identical sequences.

### CatRange predicts kinetic regimes with robust order-of-magnitude recovery

CatRange was trained to predict kinetic regimes and functional shifts by integrating enzyme sequence context with substrate chemical identity, bypassing the need for explicit structural priors (Fig. 2A). The framework leverages a 1920-dimensional joint representation—concatenating ESM-C protein language embeddings (13) and ChemBERTa molecular embeddings (14)—to drive task-specific gradient-boosted decision-tree classifiers for $k_{cat}$ and $K_{M}$ (Fig. 2; SI Appendix, Section S4). Gradient boosting was used as a stable nonlinear classifier that builds successive decision trees to refine errors from earlier trees (22, 23), allowing CatRange to learn associations between sequence context, substrate identity, and kinetic regime without imposing an explicit structural contact model. However, we compared ESM-C with ESM-2 while holding the substrate representation and classifier framework fixed. Across standard test and independent hold-out evaluations (SI Appendix, Section S3), the two enzyme representations performed similarly, whereas ESM-C showed a modest advantage in macroaveraged Matthews correlation coefficient (MCC), a balanced classification metric that incorporates all true/false positives and negatives, under the more stringent out-of-distribution (OOD) anchor and few-shot external PafA mutation settings (Fig. 3A). Because these settings most directly probe generalization under distribution shift and sparse mutation evidence, ESM-C was used as the default representation for downstream CatRange analyses.

**Figure 2:**
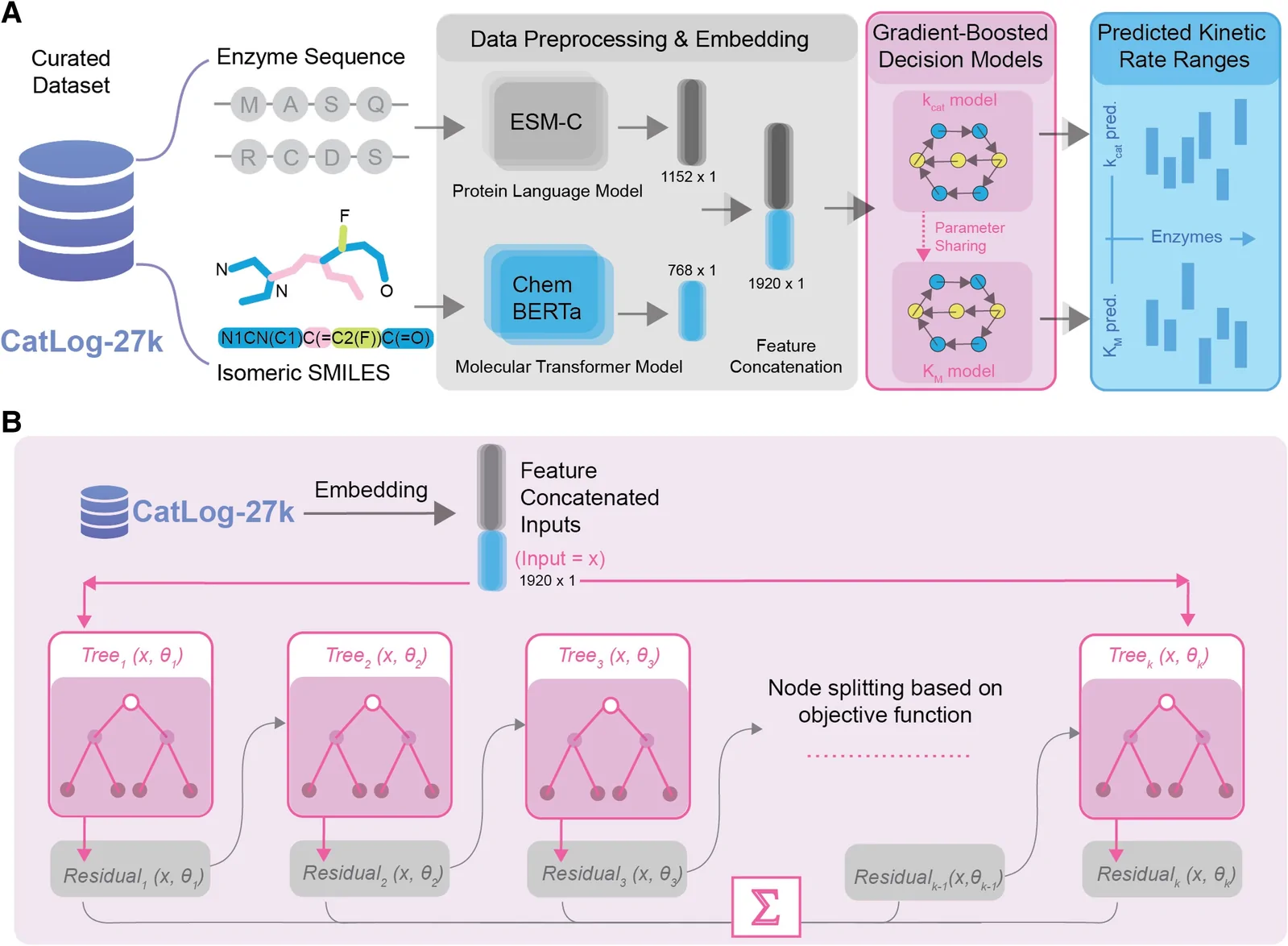
CatRange model workflow and architecture for enzyme kinetics prediction. A) CatLog-27k enzyme–substrate pairs are represented by concatenating ESM-C sequence (1152-dimensional) and ChemBERTa isomeric SMILES (768-dimensional) embeddings into a unified 1920-dimensional feature space. Separate gradient-boosted classifiers predict catalytic turnover (*k*_*cat*_) and Michaelis constant (*K_M_*) as log_10_-spaced kinetic regimes. While trained independently, the models share structural hyperparameters to maintain architectural consistency. B) Schematic of the sequential gradient-boosting mechanism used by CatRange. Trees are added sequentially to minimize classification loss, with each stage refining the ensemble's ability to assign enzyme–substrate pairs to kinetic regimes. The *k*_*cat*_ and *K_M_* models share hyperparameter settings but are trained as separate classification tasks.

**Figure 3:**
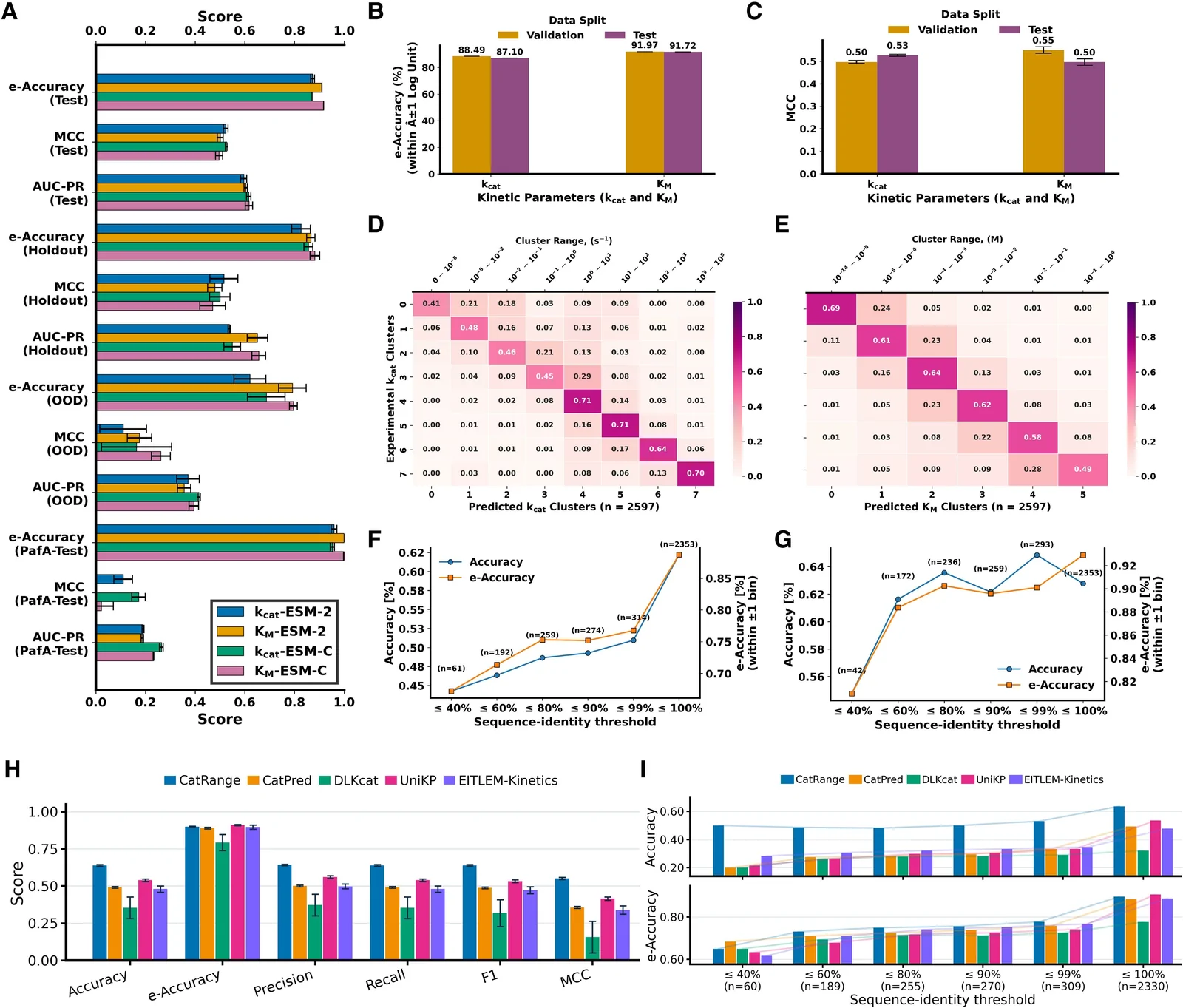
CatRange performance and comparative benchmarking. A) Evaluation of ESM-2 vs. ESM-C embeddings across stratified CatLog-27k test folds, an independent experimental holdout (*n* = 100), an OOD WT–MDAla anchor set (*n* = 44), and the PafA landscape (*n* = 310). Error bars denote cross-validation mean ± SD. B and C) Validation and test performance of the default ESM-C model for *k*_cat_ and *K_M_* evaluated by *e-accuracy* (within ±1 experimental bin) (B) and MCC (C). D and E) Row-normalized confusion matrices for *k*_cat_ and *K_M_*, respectively; rows represent experimental kinetic bins (clusters) and columns denote predicted bins. F and G) Homology-stratified exact-bin accuracy (left axis) and *e-accuracy* (right axis) for *k*_cat_ and *K_M_*, respectively, across cumulative test-to-training sequence-identity thresholds; exact-bin accuracy, e-accuracy, and sample sizes are shown. H) Bin-compatible *k*_cat_ benchmark of CatRange, CatPred, DLKcat, UniKP, and EITLEM-Kinetics across accuracy, e-accuracy, precision, recall, F1, and MCC after continuous comparator outputs were mapped to the shared kinetic-regime space. Bars show mean ± SD across the five matched test folds (source data, SI Appendix, Table S4). I) Homology-stratified *k_cat_* benchmark across cumulative sequence-identity thresholds under the same bin-compatible scoring framework. At each threshold, metrics were computed on the intersection of enzyme–substrate pair IDs with evaluable predictions from all five methods, ensuring that every model was scored on the same examples; displayed *n* values denote these common comparison sets (source data, SI Appendix, Table S5).

CatRange consistently recovered experimentally supported kinetic regimes within a ±1-bin neighborhood across cross-validation folds. Under standard fivefold cross-validation, CatRange demonstrated stable order-of-magnitude recovery. For *k*_*cat*_, *e-accuracy* (predictions falling in the exact or immediately adjacent kinetic bin) reached 87.10% on the test set; for *K_M_*, it reached 91.72% (Fig. 3B). As expected for a highly imbalanced classification task, exact class-balanced discrimination was lower but robust, with test MCCs of 0.53 for *k*_*cat*_ and 0.50 for *K_M_*, and exact-bin accuracies of 61.23 and 60.09%, respectively (Figs. 3C and S3A–D). Row-normalized confusion matrices confirmed that errors were heavily concentrated along the diagonal and neighboring bins (Fig. 3D, E), preserving the functional interpretation of log-scaled regimes even when exact-bin assignment was missed, which is very key to interpreting CatRange performance. Qualitative t-distributed stochastic neighbor embedding (t-SNE) projections further supported this, showing that predicted labels retained broad class structure in the embedded feature space rather than visually collapsing into a single dominant class (Fig. S3E, F).

CatRange's paired representation improved exact-bin and class-balanced discrimination for both kinetic parameters. Feature ablations confirmed the biological necessity of this paired formulation (SI Appendix, Section S6.1). Within the ablation framework, the full model established a baseline MCC of 0.51 for *k*_*cat*_. While sequence embeddings alone captured much of the coarse order-of-magnitude signal (e.g. *k*_*cat*_  *e-accuracy* dropped minimally from 0.87 to 0.86 without substrate inputs), substrate identity was strictly required to maximize exact-bin resolution, as the sequence-only MCC dropped substantially to 0.43. This divergence was particularly pronounced for *K_M_*, conforming to the biochemical reality that substrate-binding determinants are more chemically tractable than the complex transition-state dynamics required for catalytic turnover (Fig. S4). “Beyond component ablations, CatRange retained predictive structure across cumulative sequence-identity thresholds” (Fig. 3F, G). Although exact-bin accuracy predictably increased as more sequence-similar enzymes were included—scaling to ∼62% for *k*_*cat*_ and ∼63% for *K_M_* at the full ≤100% threshold—predictions remained highly informative in the most divergent ≤40% subsets. Specifically, at ≤40% sequence identity, CatRange maintained an exact-bin accuracy of ∼44% (*e-accuracy* ∼69%) for *k*_*cat*_ (n = 61) and ∼55% (*e-accuracy* ∼81%) for *K_M_* (n = 42).

To contextualize this performance, we benchmarked CatRange's *k_cat_* predictions against CatPred, DLKcat, UniKP, and EITLEM-Kinetics using matched datasets and a shared scoring system (SI Appendix, Section S5.2). When continuous outputs from comparator models were mapped to matched discrete kinetic-regime bins before scoring, CatRange demonstrated superior exact-bin resolution (64% accuracy, 0.55 MCC) compared to the best-performing continuous models (e.g. UniKP: 54% accuracy, 0.42 MCC), while *e-accuracy* remained comparably high (∼89–91%) across methods (Fig. 3H; Table S4). This similar e-accuracy actually masks a critical flaw in the continuous models: they default to safe, average guesses. While 70.0% of true experimental labels fell into the central kinetic ranges (bins 3–5), CatPred, DLKcat, EITLEM-Kinetics, and UniKP severely over-predicted this middle range (assigning 79.3–90.7% of predictions there), making 37.1–43.9% of their errors purely local. CatRange, however, proportionally assigned 70.3% to the center, enabling it to successfully detect rare, extreme mutations. For the sparsely represented extreme bins 1, 6, and 7, CatRange exactly recovered 59.7, 64.4, and 72.6%, substantially outperforming the highest comparator recalls (34.5–41.2%), though UniKP slightly won the heavily populated bin 4 (71.1% vs. 68.9%). This extrapolation vulnerability becomes glaring when evaluating highly unfamiliar sequences (≤40% sequence identity; Fig. 3I; Table S5). Here, true central bins comprised 58.3% of the 60-entry set; CatRange assigned a proportional 60.0% (retaining 0.500 accuracy, 0.416 MCC), whereas continuous comparators dumped 80.0–98.3% of predictions into the center, causing exact accuracies to plummet to 0.200–0.283 (MCC = 0.039–0.127). Thus, under the shared bin-compatible evaluation, CatRange more closely preserved kinetic-regime structure, with its largest advantage in exact recovery of low- and high-activity classes and in the low-sequence-identity diagnostic.

### CatRange detects mutation-driven shifts across kinetic regimes

Beyond interpolating baseline kinetic regimes, predictive models must actively detect functional shifts driven by localized catalytic substitutions. We evaluated this mutation sensitivity across four intentional and nonequivalent hierarchy of settings designed to probe distinct forms of generalization (Fig. 4; SI Appendix, Sections S3 and S7). “These settings ask whether CatRange detects mutation-associated movement across kinetic regimes rather than simply reproducing wild-type-like predictions for closely related sequences.” *First,* we established a baseline using homology-stratified mutants (≤80% sequence identity; *n* = 14), encompassing diverse substitutions outside the active site. Predictions for these mutants generally remained local to the experimentally observed *k_cat_* and *K_M_* regimes, with predicted ranges overlapping or neighboring the measured bins (Figs. 5A and S5A). Because these mutations involve diverse peripheral substitutions, this localized error structure confirms that the model's mutation response is stable under sequence-distribution shift and is not a superficial alanine-scanning artifact.

**Figure 4:**
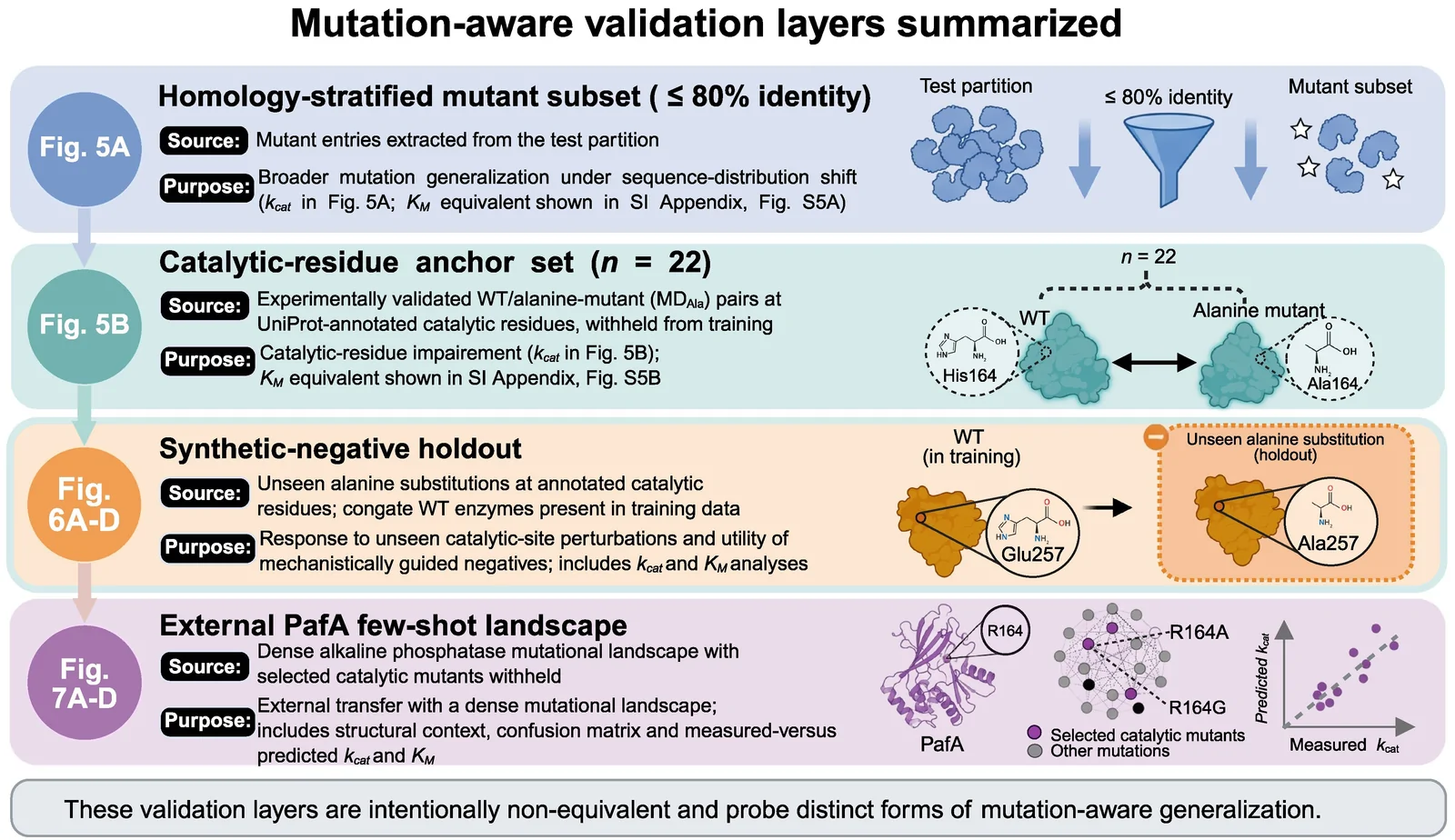
Mutation-aware validation layers. Schematic overview of the four distinct evaluation layers designed to rigorously test the model's mutational sensitivity. (Tier 1) Homology-stratified subsets test broad generalization (Fig. 5A). (Tier 2) Curated WT–MD_Ala_ anchors (*n* = 22) probe specific catalytic impairment (Fig. 5B). (Tier 3) Synthetic negatives evaluate unseen active-site perturbations (Fig. 6). (Tier 4) The dense PafA landscape tests external few-shot transfer and structural boundaries (Fig. 7).

**Figure 5:**
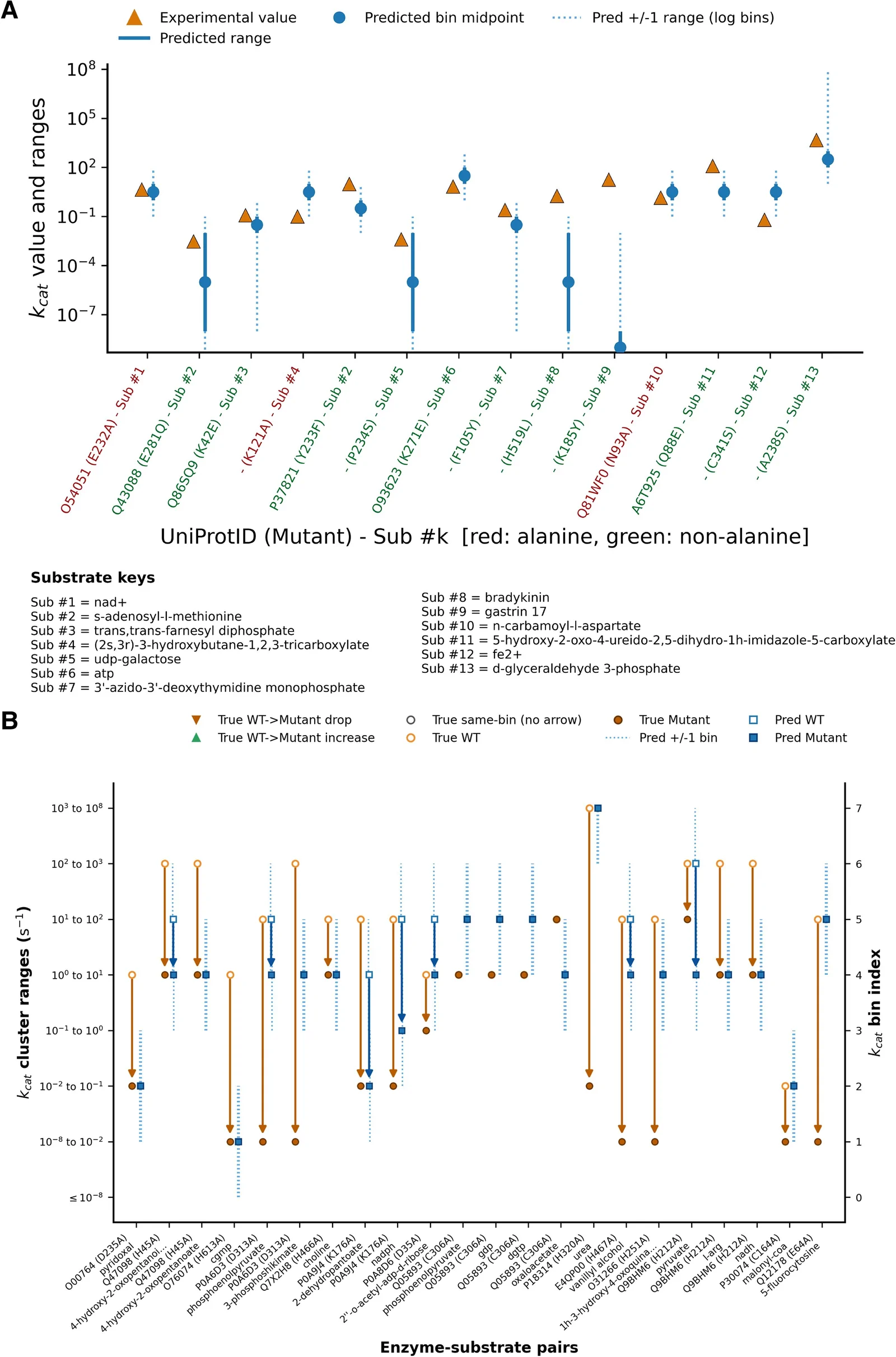
Predicting kinetic shifts across diverse mutants and catalytic-residue anchors. A) Performance on a homology-stratified mutant subset (≤80% sequence identity). Orange triangles denote continuous experimental *k*_cat_ values. Blue circles represent predicted bin midpoints, with solid lines indicating the boundaries of the exact predicted kinetic bin and dotted lines denoting the ±1 bin tolerance. The *x*-axis labels differentiate alanine (red) and nonalanine (green) substitutions. B) Kinetic regime-shift predictions for the strictly held-out and OOD catalytic-residue anchor set (*n* = 22 WT–MD_Ala_ pairs). The dual *y*-axis maps continuous *k*_cat_ ranges (left) to discrete prediction bins (right). Arrows trace the directional shift from the WT enzyme (open markers) to the alanine mutant (filled markers). Comparing experimental drops (orange) against CatRange predictions (blue) demonstrates the model's ability to detect localized functional impairment rather than defaulting to WT assignments for highly homologous sequences. The corresponding *K_M_* anchor analysis is shown in Fig. S5.

We next challenged the model with experimentally curated WT–MD_Ala_ OOD anchors (*n* = 22 pairs), a stringent extrapolation test of direct catalytic-residue perturbation strictly withheld from training (SI Appendix, Section S2.2). Across these paired comparisons, CatRange often reproduced the direction and approximate magnitude of WT-to-mutant shifts, with many mutant predictions falling within the ±1-bin tolerance window, as defined by e-accuracy (Figs. 5B and S5B). Longitudinal trajectories effectively tracked experimental curves, with predicted trajectories closely tracking experimental curves and remaining largely within the ±1-bin tolerance bands for both parameters (Fig. S7). Importantly, CatRange reflected the natural heterogeneity of these mutations, where some substitutions decimated *k_cat_*, others primarily shifted *K_M_*, and some caused negligible change, avoiding the artifactual collapse of all mutations to a uniform inactive state. When misclassifications occurred, the model overwhelmingly defaulted toward the WT bin rather than spuriously overestimating catalytic loss. This conservative fallback behavior underscores the challenge of extrapolating massive mutational penalties in OOD regimes without explicit structural context.

To isolate active-site sensitivity, a synthetic-negative hold-out (*n* = 10) tested the model on unseen catalytic-site alanine variants whose cognate WT backgrounds were present during training. While active-site alanine substitutions can occasionally yield rate enhancements, such as relieving steric hindrance for non-native substrates (e.g. active-site expansion in cytochrome P450s (24) or “bump-hole” kinase engineering (25)), our curated WT–MD_Ala_ calibration anchors strictly captured functional impairment. To avoid extrapolating beyond this empirically calibrated distribution, we evaluated the model's response using binary directional labels restricted to these observed failure modes (Drop vs. No-Drop for *k*_*cat*_; Increase vs. No-Increase for *K_M_*). These expected shifts were explicitly calibrated by a meta-learner using physicochemical substrate properties and BLOSUM62 mutational severity scores derived directly from our experimental WT–MD_Ala_ anchor pairs (SI Appendix, Section S2.3). Evaluated against these mechanistically informed and calibration-derived reference labels, CatRange achieved high specificity for capturing expected *k*_*cat*_ decreases (precision = 1.00, recall = 0.78, F1 = 0.87, MCC = 0.51) (Figs. 6C and S6A). For *K_M_* increases, performance was positive but attenuated (MCC = 0.50), reflecting the biochemical reality that apparent-affinity shifts are highly context-dependent and harder to reliably predict from sequence alone (Figs. 6D and S6B). Crucially, while WT predictions for both *k*_*cat*_ and *K_M_* remained firmly anchored to experimental values (Fig. 6A, C), the corresponding unseen alanine variants cleanly shifted away where necessary from their cognate WT regimes in the empirically expected direction (Fig. 6B, D).

**Figure 6:**
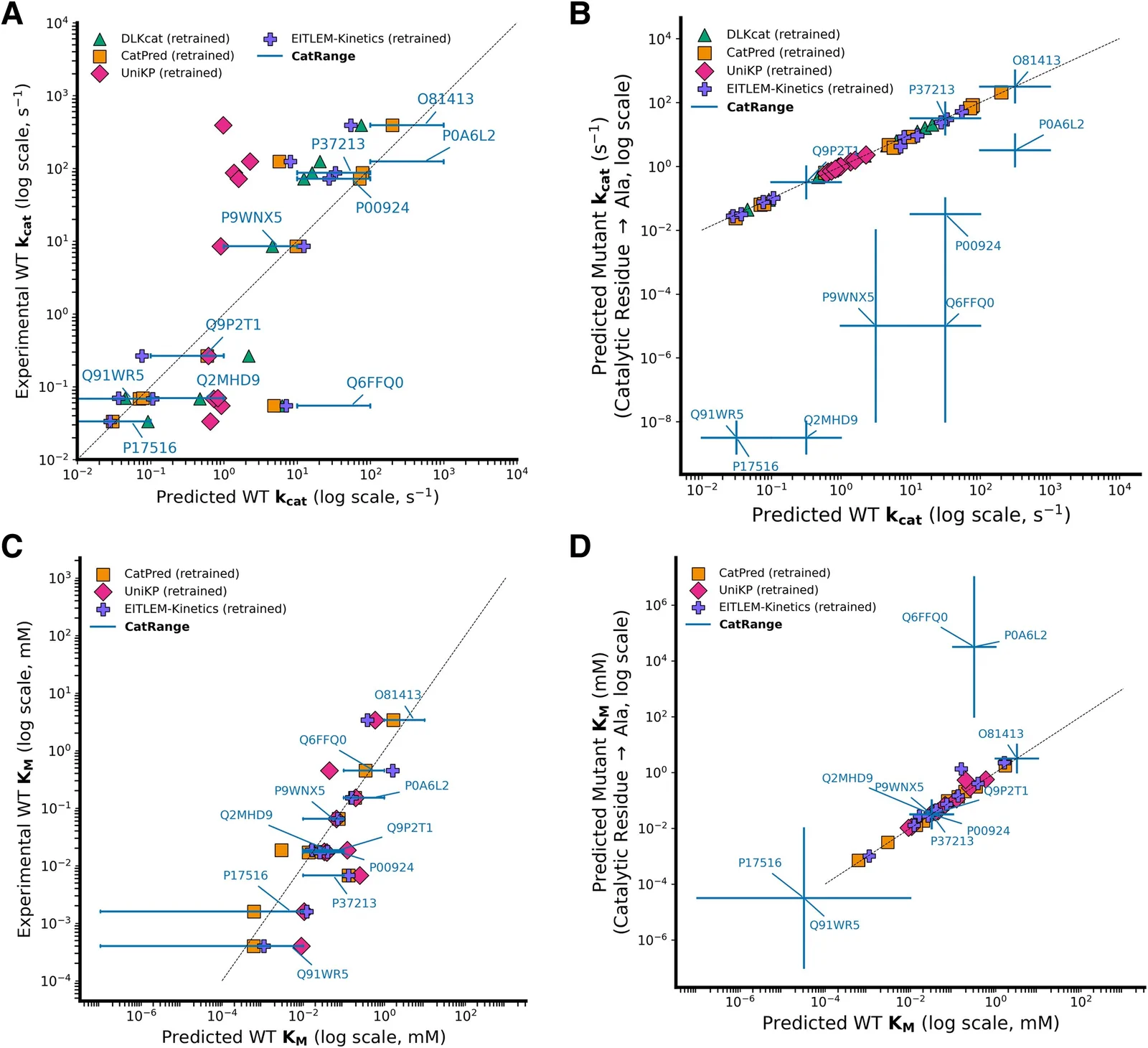
Evaluating mutation sensitivity on unseen catalytic-site variants. A and C) Experimental vs. predicted WT parameters for *k*_cat_ (A) and *K_M_* (C). Retrained continuous benchmark models yield point predictions, whereas CatRange outputs discrete log-scaled kinetic ranges, represented by blue crosshairs. Because cognate WT sequences were present during training, all models successfully anchor predictions near the experimental identity line (dashed). B and D) Predicted mutant vs. predicted WT parameters for *k_cat_* (B) and *K_M_* (D). The dashed diagonal line (y=x) represents mutation blindness, where a model assigns identical predictions to highly homologous sequences. While the continuous benchmark models collapse onto this identity line—defaulting to WT-like predictions—CatRange correctly shifts away from the diagonal to capture expected functional impairment (decreased *k*_cat_, increased *K_M_*). DLKcat is omitted from *K_M_* panels as it exclusively predicts catalytic turnover.

To definitively establish the necessity of these curated variants, we trained a strict ablation model omitting all synthetic negatives (SI Appendix, Section S6.2). We focused this ablation specifically on *k*_*cat*_ predictions, as active-site ablations fundamentally govern transition-state turnover rather than strictly dictating substrate affinity. The ablated model's expected-drop recall collapsed to 0.044, the mean predicted WT-to-mutant bin shift vanished from 2.12 to 0.14 bins, and the average number of predicted drop calls decreased from 8.0 to 0.4 out of 10 (Fig. S8B). Furthermore, on the independent OOD (WT–MD_Ala_) anchor set, the full CatRange model improved mutant-bin recovery relative to the ablation (Fig. S8C). These results indicate “calibrated catalytic-site variants act as essential contrastive examples for sensitivity to catalytic-site perturbations in the evaluated settings”. Taken together, these evaluations demonstrate that CatRange actively predicts movement across kinetic regimes when active-site chemistry is perturbed. Simultaneously, the model's conservative fallback behavior defines the limits of the framework: CatRange is designed for order-of-magnitude regime prediction, not sub-bin ranking or fine-grained mechanistic attribution.

### Dense mutational landscapes define the structural boundaries of sequence-based inference

While curated anchors and synthetic variants provide targeted tests of active-site sensitivity, understanding a model's operational boundaries requires mapping its performance across a complete, densely sampled mutational landscape. As the final layer of our validation hierarchy, we evaluated CatRange on PafA from *Elizabethkingia meningoseptica*, originally reported by Markin et al. (26). Utilizing a high-throughput dataset of over 1,000 assayed single-site variants (glycine, valine, and alanine) assayed against carboxy 4-methylumbelliferyl phosphate ester. From this landscape, we established a strictly held-out test partition of 310 variants. This few-shot setting tests the model's ability to track how local substitutions redistribute apparent kinetic parameters (*k*_*cat*_ and *K_M_*) within a single, fixed structural scaffold rather than extrapolating across diverse enzyme families (SI Appendix, Section S8.1; Fig. 7A).

**Figure 7:**
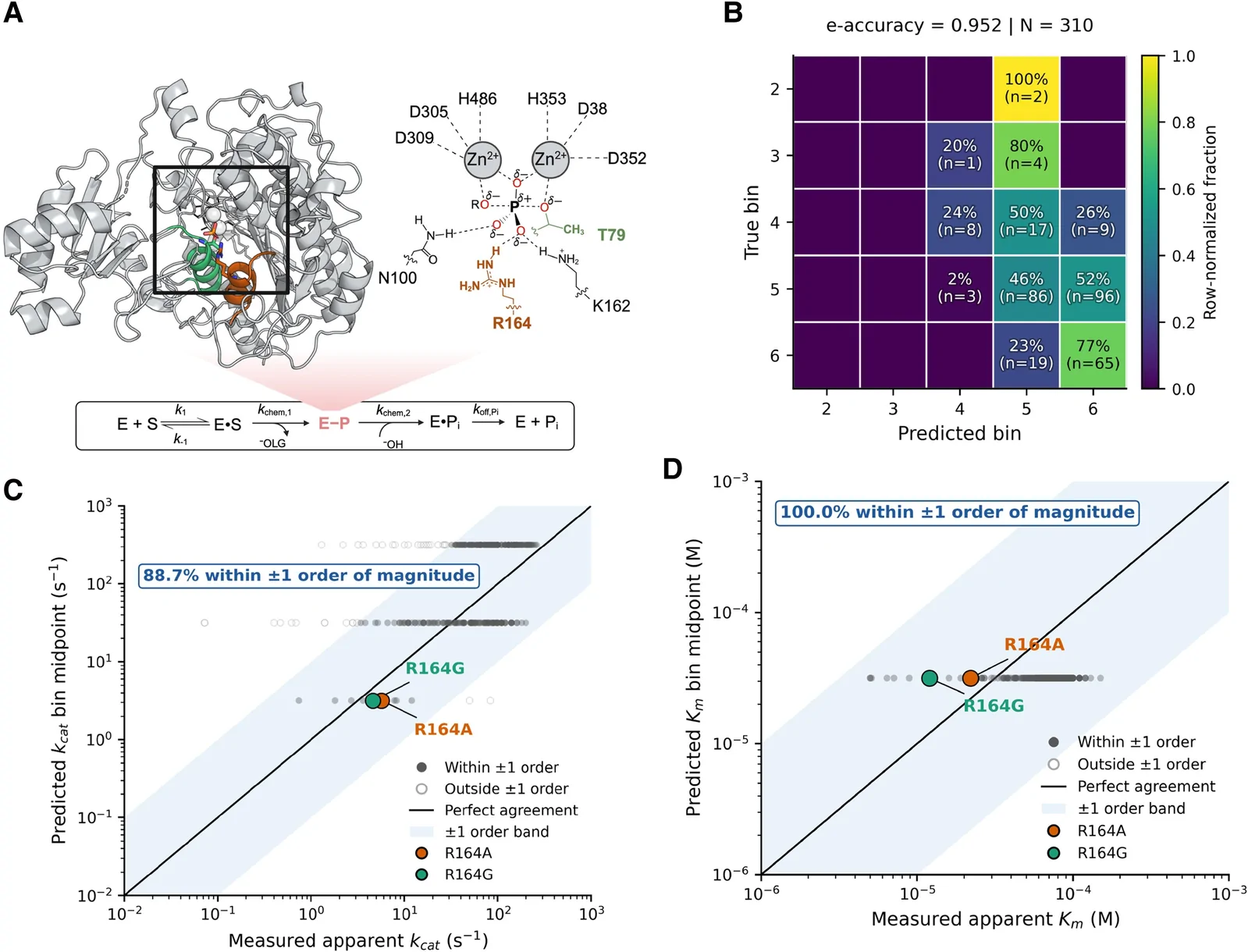
Evaluating mutational sensitivity and structural boundaries within a dense, single-enzyme landscape. A) Structural and mechanistic context of PafA, highlighting active-site coordination and the critical R164 residue. B) Row-normalized confusion matrix for the 310 held-out *k*_cat_ variants. Aggregate e-accuracy was 95.2%, with recovery strongly localized within adjacent log-scaled kinetic regimes. C and D) Continuous-scale evaluation comparing measured apparent *k*_cat_ (C) and *K_M_* (D) to the geometric midpoints of the predicted discrete bins. The shaded band denotes a 10-fold tolerance around perfect agreement. Solid markers denote variants successfully recovered within this biological variance window. Conversely, open circles denote severe-shift variants that fall outside this band, explicitly exposing the structural boundaries of sequence-based inference when substitutions disrupt complex, highly coordinated 3D environments. While the specific single-mutant records for R164A and R164G were withheld from training, an R164A-containing double mutant was present; their successful recovery therefore illustrates CatRange's capacity to extrapolate localized catalytic perturbations.

CatRange maps continuous experimental measurements into discrete, log-scaled bins, where a single bin shift represents a full order of magnitude—a 10-fold change in actual catalytic turnover. Within this framework, the 310-variant *k*_*cat*_ test set was heavily concentrated in the central kinetic ranges, spanning nearly four orders of magnitude, reflecting the biological reality that most random point mutations induce moderate rather than catastrophic functional shifts. True bin 5 alone accounted for 59.7% of the held-out data (Fig. 7B). Consequently, while CatRange achieved 51.3% exact-bin accuracy and 95.2% aggregate *e-accuracy*, this metric must be interpreted rigorously: rather than demonstrating uniform discrimination across all theoretical activity extremes, the high aggregate score reflects robust local recovery in the dominant, biologically populated bins. Adjacent-bin recovery rates across true bins 2 through 6 were 0, 20.0, 73.5, 100, and 100%, respectively. Crucially, the confusion matrix confirms that when predictions missed the exact bin, errors were largely confined to neighboring log-scaled classes rather than spuriously scattered, preserving the model's utility for regime-level prioritization (SI Appendix, Section S8.2).

Continuous-scale evaluations provided a complementary, physical assessment of these regime predictions. When mapped to predicted bin geometric midpoint in log space, 88.7% of the held-out variants fell within one order of magnitude of their measured apparent *k*_*cat*_ (Fig. 7C). Because this subset included alanine, glycine, and valine substitutions, the performance extends beyond simple alanine-scanning behavior. To examine active-site sensitivity within this dense sequence neighborhood, we evaluated the R164A and R164G single substitutions. While these specific single-mutant records were absent from training, the N100A/R164A double mutant was included in the prespecified training subset (SI Appendix, Section S8.1). We therefore treat the successful recovery of R164A and R164G within the ±1 order-of-magnitude band as illustrative held-out cases of single-mutant extrapolation for the purview of this study (Figs. 7C and S9). For apparent *K_M_*, all held-out measurements fell within one order of magnitude of the predicted midpoint (Fig. 7D); however, because the PafA *K_M_* measurements occupy a narrower range (∼1.6-order-of-magnitude) for a single substrate, this serves primarily as a supportive baseline control rather than a stringent test of affinity-shift detection.

Critically, this landscape exposed the physical limitations of sequence-based kinetic extrapolation at the low-activity tail. Variants with severe, experimentally measured *k*_*cat*_ losses were frequently predicted closer to the well-populated central ranges. This reflects a conservative failure mode: the model underestimates large deleterious shifts rather than unrestrictedly predicting catastrophic loss-of-function (Fig. S9). Markin et al. (26) noted that many of the most severe apparent *k*_*cat*_ reductions in this in vitro dataset frequently stem from global kinetic folding traps rather than localized catalytic ablation. Post hoc structural inspection revealed that several of these underestimated variants (e.g. G56, D144, L297, F332, G465, and H486) inhabit locally dense or structurally constrained environments. Residue H486, for example, is highly networked with 18 neighboring residues within the structural contact threshold (Fig. S10). Substitutions at these highly coordinated positions likely propagate severe, nonlocal geometric disruptions through the architecture, exposing the boundaries of what sequence representations can infer without explicit 3D context. These failure modes definitively delimit the resolution of the current sequence-based framework. The ±1-bin prediction tolerance directly mirrors the inherent, order-of-magnitude experimental variance routinely observed when identical enzyme–substrate pairs are assayed across different studies (Fig. S2). Within this realistic biological variance window, CatRange successfully recovers most held-out variants and detects direct active-site perturbations. However, it correctly yields to explicit structural modeling for the precise mechanistic attribution of complex allostery, establishing “CatRange as a robust, regime-level prioritization tool that maintains an honest boundary around structural resolution”.

## Discussion

CatRange establishes a mutation-aware framework for enzyme kinetics prediction by integrating rigorous biochemical data discipline with an evaluation strategy explicitly matched to the inherent uncertainty of experimental enzymology. By reformulating *k*_*cat*_ and *K_M_* prediction as classification into log-scaled kinetic regimes, CatRange shifts the computational paradigm from brittle pointwise regression to biologically robust order-of-magnitude inference. CatLog-27k provides the foundation for this framework. By reconciling kinetic measurements across public resources, consolidating duplicate records via median pooling rather than extreme-value selection, and introducing calibrated catalytic-site alanine variants, this dataset directly addresses two persistent bottlenecks in the field: sensitivity to noisy public measurements and the failure of existing models to penalize localized functional impairment (9, 12). This approach reflects a fundamental principle of biological function learning: contrastive negative evidence is required to distinguish true catalytic impairment from nominal WT sequence similarity (27, 28).

This regime-based formulation is central to CatRange's biophysical interpretation. Enzyme kinetic values inevitably vary across independent studies due to differences in pH, temperature, buffer composition, and measurement protocol, making exact numerical agreement an unstable target for massive, aggregated datasets (1, 29, 30). For instance, according to criteria from Bar-Even et al. (30), *K_M_* values can be categorized into high (>1 mM), moderate (0.1–1 mM), and low (≤0.1 mM) clusters, distinguishing enzymes that maintain metabolic flux across dynamic conditions from those requiring high substrate affinity. Similarly, *k*_*cat*_ values span high (>1,000 s^−1^), moderate (100–1,000 s^−1^), and low (≤100 s^−1^) categories, reflecting known variation in catalytic efficiency. Furthermore, nominal in vitro parameters often diverge from the effective kinetic behavior realized in vivo due to cellular regulatory states and metabolite availability (31, 32). CatRange therefore prioritizes stable, order-of-magnitude recovery, rewarding predictions that fall within the exact experimental bin or an adjacent log-scaled regime (*e-accuracy*). This tolerance ensures that the model successfully recovers functional kinetic neighborhoods without overfitting to assay-specific experimental noise. Crucially, this framework recontextualizes conservative errors: when predictions fall near the WT regime rather than extrapolating to extreme loss-of-function, they act as informative boundaries, defining where sequence-based inference ends and where explicit structural modeling must begin.

The paired enzyme–substrate representation is likewise a biochemical necessity rather than an architectural embellishment. Catalytic turnover and apparent affinity are conditional properties of an enzyme acting on a specific molecular target. While sequence embeddings alone can capture a coarse order-of-magnitude baseline activity (i.e. *k*_*cat*_), substrate identity is required to build enzyme class/reaction-level awareness and binding (*K_M_*) kinetics. CatRange's performance arises from modeling this paired context. Yet, this representation remains deliberately lightweight: by bypassing explicit 3D physical contacts and conformational ensembles, the model achieves high scalability across the >20k-entry landscape while maintaining a transparent ceiling on its mechanistic resolution. Importantly, structural information should not be added indiscriminately: related work, including CatPred (9), suggests that naive structural integration can degrade performance when structural context is not mechanistically aligned with the prediction task.

Mutation sensitivity is the defining capability of CatRange. Across OOD catalytic anchors, broader mutant subsets, and the dense PafA variant landscape, the model actively tracks mutation-associated movement across kinetic regimes. However, the PafA analysis also clearly delineates the physical limits of sequence-based embeddings. By underestimating severe *k*_*cat*_ losses at highly networked or flexible positions, the model reveals that even mutation-aware language models cannot spontaneously infer the catastrophic nonlocal geometric disruptions caused by specific structural mutations. However, the practical utility of CatRange lies in generating interpretable, actionable kinetic hypotheses. In enzyme engineering and synthetic biology, regime-level predictions can prioritize active variants and filter catastrophic losses prior to costly screening. In biomedical contexts and pharmacogenomics, CatRange can flag sequence variants of uncertain significance that are likely to disrupt metabolic flux (33, 34). In systems biology, bounded order-of-magnitude estimates for *k*_*cat*_ and *K_M_* can directly parameterize enzyme-constrained metabolic models (35–37). By narrowing the parameter search space, especially when relying on stochastic surrogates, CatRange explicitly improves the interpretability of flux predictions (38–40). Across these domains, CatRange serves as a highly robust prioritization tool, aligning computational capability with the practical realities of experimental enzyme kinetics.

## Limitations of the study

CatRange must be interpreted within the compositional and representational boundaries of its foundational data. *First,* while CatLog-27k is rigorously curated, it inherits the historical reporting biases of enzymology. Well-characterized classes such as hydrolases, transferases, and oxidoreductases are heavily represented, whereas translocases and rare catalytic mechanisms remain sparse, inherently limiting the model's coverage of the global functional space. *Second,* the framework is constrained by its lightweight molecular representations. Substrate embeddings derived from isomeric SMILES capture graph-level chemical identity and stereochemistry but remain blind to 3D electrostatics, solvent exposure, and induced-fit conformational dynamics. Similarly, pooled protein language embeddings (ESM) provide scalable sequence descriptors but lack explicit positional contact density. Consequently, while CatRange successfully tracks local chemistry perturbations (e.g. active-site alanine substitutions), it struggles to precisely quantify the magnitude of functional collapse driven by long-range allosteric coupling or disrupted local packing. *Third,* the synthetic catalytic-site alanine variants, while essential for exposing the model to localized impairment, do not capture the full spectrum of mutational outcomes. Alanine substitution effectively models the removal of side-chain chemistry, but it cannot represent compensatory epistatic substitutions, complex context-dependent activity enhancements, or gain-of-function mutations—such as steric relief, that occasionally increases *k*_*cat*_ for bulky non-native substrates when large active-site residues are truncated (such as seen in 4-coumaryl CoA ligase (41)). Expanding beyond these limitations will require the integration of experimentally anchored, multisubstrate mutant landscapes containing both deleterious and gain-of-function substitutions. *Finally,* while the order-of-magnitude classification strategy successfully absorbs assay heterogeneity including reporting variability of enzyme kinetics across pH, ionic strength, temperature, enzyme source, construct design, and measurement protocol (26, 29–31), it intrinsically trades away sub-bin precision. Variants with nuanced differences in side-chain chemistry that cause subtle intrabin kinetic shifts may be mapped to the identical regime. Future refinements seeking exact pointwise mechanistic ranking will require the targeted integration of catalytically relevant 3D structural subcontexts to resolve the subtle geometric and dynamic effects that sequence and graph embeddings alone cannot capture. Reaction-core resources such as RC-Hydrolase, which we have put forward as a recipe to collate catalytically relevant substructures for >19,000 experimentally resolved hydrolases, provide one route toward incorporating active-site architecture in a targeted and interpretable manner (42). These directions are likely to improve CatRange most in the regimes where it currently struggles, such as—severe destabilizing mutations, structurally dense residues, nonalanine substitutions, and condition-dependent kinetic effects.

## Materials and methods

All methods used in this study are detailed in SI Appendix, Supplementary Methods and Analyses, including database construction and curation, kinetic-regime definition, enzyme and substrate embedding generation, construction and calibration of catalytic-site variants, model development and hyperparameter optimization, data partitioning and cross-validation, homology-stratified and mutation-focused validation, ablation and benchmark analyses, external case-study evaluation, and statistical analyses.

## Supplementary Material

pgag309_Supplementary_Data

## Data Availability

CatRange source code, analysis scripts, data, and documentation are available at https://github.com/ssbio/CatRange and https://github.com/TKAI-LAB-Mali/CatRange. The CatLog dataset is available at https://chowdhurylab.github.io/tools/catlog-latest.html. Model inference is accessible through web interfaces at https://catrange.structf.studio, https://catrange.sahassbio.com, https://predictor.openkinetics.org, and through an open-source Python Google Colab notebook. The inference interfaces utilize CLEAN as a preliminary filter to determine if a submitted protein sequence is an enzyme. If a sequence is classified as a nonenzyme, it is retained in the output dataset, but its downstream CatRange kinetics predictions are marked as skipped. Code for the earlier RealKcat implementation, which assigned synthetic negative examples to a designated inactive class (“Cluster 0”), is available at https://github.com/TKAI-LAB-Mali/CatRange.
