# Supplementary material for: CatRange enables robust prediction of enzyme variant kinetic regimes
Source: PNAS Nexus. 2026 Sep 16;5(10):pgag309. doi: 10.1093/pnasnexus/pgag309 (PMC13659915; doi:10.1093/pnasnexus/pgag309)
Supplement: Supplementary file 1 [file pgag309_supplementary_data.zip › PNASNEXUS-PNASNEXUS-2025-01944RR-s01.pdf]

PNAS nexus

Supplementary Information for

CatRange Enables Robust Prediction of Enzyme Variant Kinetic Regimes

Karuna Anna Sajeevan[1,3,4,5,*], Abraham Osinuga[2,*], Arunraj B[1], Sakib Ferdous[1], Nabia Shahreen[2], Mohammed Sakib Noor[1], Shashank Koneru[1], Laura Mariana Santos-Correa[1], Rahil Salehi[1], Niaz Bahar Chowdhury[2], Randy Aryee[1,3], Brisa Calderon-Lopez[1], Supantha Dey[1], Ankur Mali[6], Rajib Saha[2,†], and Ratul Chowdhury[1,3,4,†]

[1]Department of Chemical and Biological Engineering, Iowa State University, Ames, Iowa, USA

[2]Department of Chemical and Biomolecular Engineering, University of Nebraska-Lincoln, Lincoln, Nebraska, USA

[3]The Center for Biorenewable Chemicals, Iowa State University, Ames, Iowa, USA

[4]Ames National Laboratory, Ames, Iowa, USA

[5]Srinivasa Ramanujan Institute for Basic Sciences, Pi Hills, 8th Mile, Velloor, Pampady, Kottayam, Kerala, India

[6]Department of Computer Science and Engineering, University of South Florida, Tampa, Florida, USA

*Karuna Anna Sajeevan and Abraham Osinuga share first authorship.

[†]Email all correspondences to: rsaha2@unl.edu or ratul@iastate.edu

The PDF file includes:

Supplementary Methods and Analyses
Figures S1 to S10
Tables S1 to S2
SI References

Other supplementary materials for this manuscript include the following:

Supplementary Tables S3 to S5

SI Appendix

Contents

1. CatLog-27k Construction and Curation 4
1.1 Data sources and record harmonization 4
1.2 Manual literature reconciliation and quality control 4
1.3 Duplicate consolidation, median pooling, and dataset composition 5
1.4 Sequence-identity visualization of CatLog-27k 5
1.5 Agent-assisted CatLog framework 5
2. Kinetic-Range Definition and Mutation-Aware Supervision 6
2.1 Log-scaled kinetic ranges and prediction bins 6
2.2 Literature-derived WT–MD_{Ala} catalytic-residue anchors 6
2.3 Meta-learning calibration of mutation-effect labels 7
2.4 Generation and labeling of synthetic catalytic-site alanine variants 7
3. Data Partitioning and Leakage-Controlled Evaluation Design 8
3.1 Standard cross-validation and independent holdout 8
3.2 Synthetic alanine-variant partitioning 8
3.3 Mutation-focused holdouts 8
3.4 Homology-stratified evaluation subsets 9
4. CatRange Model Development and Training 9
4.1 Enzyme sequence representations 9
4.2 Substrate molecular representation 9
4.3 Paired enzyme–substrate feature construction 9
4.4 Feature standardization 10
4.5 Gradient-boosted classifier training 10
4.6 Hyperparameter optimization and parameter sharing 10
4.7 Class balancing, sample weighting, and hard-example reweighting 10
5. Evaluation Metrics and Comparative Benchmarking 11
5.1 Exact-bin, e-accuracy, and class-balanced metrics 11
5.2 Comparator retraining and common-bin evaluation 11
5.3 Homology-stratified benchmark evaluation 12
6. Ablation Analyses 12
6.1 Paired enzyme–substrate representation ablation 12
6.2 Synthetic catalytic-site alanine supervision ablation 12

7. Mutation-Aware Validation 12
7.1 Homology-stratified experimental mutant subset...13
7.2 Literature-derived WT–MD_{Ala} catalytic-residue anchor set 13
7.3 Catalytic-site alanine holdout 13
8. PafA Few-Shot Mutational Landscape 14
8.1 Dataset and few-shot partitioning 14
8.2 Evaluation metrics and baseline interpretation 14
8.3 Residue-wise kinetic context 14
8.4 Post hoc structural-context analysis of failure cases 15
9. Code, Data, and Computational Resources 15
Supplementary Figures..17
Supplementary Tables 31
SI References...34

1. CatLog-27k Construction and Curation

1.1 Data sources and record harmonization

Enzyme kinetic measurements for k_{cat} and K_M were assembled from BRENDA (release 2023_1) (*1*) and SABIO-RK (May 2024 snapshot) (*2*) using custom Python workflows. The initial extraction contained 237,487 BRENDA records and 77,663 SABIO-RK records. Records were parsed for UniProt identifier, organism, EC number, substrate, mutation annotation, literature identifier, and kinetic value; entries lacking fields required for downstream reconciliation were removed. This first-pass filtering yielded 96,782 BRENDA records and 54,906 SABIO-RK records. Figure S1 summarizes the complete upstream assembly workflow.

Substrate identities were standardized through PubChem (*3*). Substrate names were queried to obtain an InChI intermediate before conversion to a standardized isomeric SMILES representation with RDKit. This route was used because multiple noncanonical SMILES strings can represent the same compound. Enzyme sequences were retrieved from UniProt (*4*) when a UniProt identifier was available; otherwise, sequence retrieval used EC number and organism information from BRENDA and UniProt, followed by application of the reported mutation annotation to reconstruct the assayed sequence. Before manual literature reconciliation, this workflow produced 33,442 k_{cat} records, 48,103 K_M records, and 26,244 raw entries containing both kinetic parameters, including 10,244 mutant records linked to 2,158 unique source articles.

The Fig. S1 workflow contains two distinct consolidation stages that should not be conflated. During raw database assembly, within-source duplicate handling reduced repeated database records before merging. After literature reconciliation, the final model-ready representation was instead constructed by grouping identical enzyme-sequence–isomeric-SMILES pairs and taking the median kinetic value across repeated measurements, as described in Section 1.3.

1.2 Manual literature reconciliation and quality control

The 2,158 source articles associated with mutant records were reviewed directly to reconcile database entries with the primary literature. The review recovered 11,175 mutant records with both kinetic parameters, resolved 1,804 inconsistencies, and added 1,072 records that were absent or incompletely represented in the aggregated database tables. Corrections involved K_M values (788 instances), 618 k_{cat} values, 240 substrate assignments, and 18 mutation annotations; additional removals are summarized in Table S1. After accounting for 140 removed or duplicate records, the 1,072 additions correspond to the net increase of 932 entries shown in Fig. S1. The curated record-level collection therefore contains 16,001 wild-type and 11,175 mutant entries, totaling 27,176 records.

The literature review also corrected parameter swaps and transcription errors that would otherwise propagate directly into model labels. For human m-NAD-malate dehydrogenase D102E in the reported fumarate-to-pyruvate context, the reconciled values were $k_{cat} = 60.61\ s^{-1}$ and $K_M = 0.00091$ M, replacing database values of 44.14 s^{-1} and 0.00255 M. For Lactobacillus pentosus D-lactate dehydrogenase Y52A with 2-ketocaproate, the reconciled values were $k_{cat} = 102\ s^{-1}$ and $K_M = 0.0007$ M, replacing 0.7 s^{-1} and 0.102 M. These examples illustrate why primary-source verification was performed before pooling and model development.

1.3 Duplicate consolidation, median pooling, and dataset composition

After reconciliation, repeated measurements were consolidated at the enzyme–substrate level by grouping identical enzyme sequences with identical isomeric SMILES and taking the median of the available k_{cat} and K_M measurements for each group. This final pooling step was chosen to reduce the influence of assay-specific extremes while retaining a representative central value (5). The underlying record-level measurements, literature provenance, and available assay metadata were preserved in the public CatLog-27k release; median pooling was applied only to derive the model-ready representation.

Repeated measurements remained highly heterogeneous even after identifier and literature reconciliation. Among the 50 sequence–substrate pairs with the largest spread, reported values for the same pair span as much as 6–8 orders of magnitude across studies (Fig. S2). This residual variation is consistent with differences in pH, temperature, buffer composition, co-substrate concentration, enzyme source, construct design, and reporting context. Median pooling yielded 24,517 unique sequence–substrate entries: 14,709 wild-type and 9,808 mutant entries. These pooled entries form the experimentally measured basis for CatRange model development.

1.4 Sequence-identity visualization of CatLog-27k

For model development, enzyme sequences longer than 1,024 residues were excluded to maintain compatibility with the protein-language-model input limit. Sequence relationships among the retained unique enzymes were visualized using an all-vs-all global-alignment workflow. The graph-generation implementation standardized retained sequences to a fixed length of 1,024 characters by padding shorter sequences and encoded characters by ordinal value. Global alignments were generated with Biopython pairwise2.align.globalxx, which implements a Needleman–Wunsch-style global alignment with unit match scores and zero mismatch and gap penalties. For each aligned pair, sequence identity was calculated as the number of identical aligned positions divided by the length of the longer unaligned sequence, $I_{ij} = N_{\text{match}} / \max(L_i, L_j)$. The resulting identity values were converted to pairwise dissimilarities as $d_{ij} = 1 - I_{ij}$.

A weighted pairwise sequence-identity graph was constructed by assigning each unique enzyme sequence as a node and using this dissimilarity, d_{ij}, as the edge cost between sequence pairs. A minimum spanning tree (MST) was then computed from this graph with NetworkX using the pairwise dissimilarity as the spanning-tree weight. The MST was used to organize the node layout: Graphviz dot was used when available, with a weighted Kamada–Kawai layout as the fallback. The final visualization retained the pairwise graph edges at these MST-derived node positions and colored each sequence by the first digit of its annotated EC number (main-text Fig. 1C). Accordingly, Fig. 1C is interpreted as an MST-organized sequence-identity network rather than as a quantitative embedding of sequence space; the plotted spatial distances are not assumed to scale directly with sequence divergence. The visualization reveals extensive intermixing of primary EC classes together with localized class enrichment in portions of the network.

1.5 Agent-assisted CatLog framework

The current CatLog-27k release was curated manually. In parallel, an agent-assisted framework was developed to standardize future updates. The framework retrieves candidate kinetic measurements, maps source records into a common schema, preserves original database fields and literature identifiers,

and flags inconsistencies in enzyme identity, organism assignment, sequence or mutation state, substrate annotation, assay context, and citation evidence. Flagged records are routed for review against retained source records, UniProt, GenBank, PubMed, and archived paper evidence. Records that remain unresolved are retained for follow-up rather than automatically forced into the curated release.

CatLog is publicly accessible at https://chowdhurylab.github.io/tools/catlog-latest.html. The database stores individual curated measurements alongside their associated metadata, representing the kinetic landscape prior to the median pooling and binning steps used to generate the CatRange training partitions. These unpooled measurements are fully retained in CatLog and included in the primary study data release. When reported by the original source, the catalog captures comprehensive assay contexts—including temperature, pH, organism, substrate, and sequence or mutation details—as well as cross-references to external databases and primary literature. To facilitate data exploration, the web interface features an instructional guide, enabling users to seamlessly search, filter, inspect individual measurements and their provenance, and download custom datasets.

2. Kinetic-Range Definition and Mutation-Aware Supervision

2.1 Log-scaled kinetic ranges and prediction bins

CatRange reformulates continuous kinetic measurements into discrete, log-scaled regimes—featuring decade-spaced interior bins and broader edge boundaries—to capture biological variance at order-of-magnitude resolution. All k_{cat} values were converted to s^{-1} and all K_M values to M before bin assignment. The k_{cat} bin edges were [0, 10^{-8}, 10^{-2}, 10^{-1}, 10^{0}, 10^{1}, 10^{2}, 10^{3}, 10^{8}] s^{-1}, and the K_M bin edges were [10^{-14}, 10^{-5}, 10^{-4}, 10^{-3}, 10^{-2}, 10^{-1}, 10^{4}] M. The broad edge intervals retain very low and very high measurements without compressing them into the central order-of-magnitude ranges (main-text Fig. 1F–H). Each predefined numerical range is treated as one discrete prediction bin.

The same bin definitions are used throughout CatRange training, benchmark conversion, mutation-effect labeling, and evaluation. This common target space is important because the WT–mutant calibration and the downstream classifier must interpret a bin shift in the same way. Exact-bin and adjacent-bin evaluation are defined in Section 5.1.

2.2 Literature-derived WT–MD_{Ala} catalytic-residue anchors

Mutation-aware supervision was anchored to 22 literature-derived *in vitro* wild-type–alanine-mutant (WT-MD_{Ala}) enzyme–substrate pairs in which alanine substitution occurred at UniProt-annotated catalytic residues (Table S3). These measurements were drawn from the curated CatLog-27k evidence base; no new kinetic measurements were generated for this study. The pairs span substantial turnover decreases, K_M increases, and cases that remain within the same kinetic bin, providing empirical examples of the heterogeneous consequences of catalytic-site alanine substitution.

The 22 pairs play two distinct roles that are kept separate in the analysis. First, they provide the experimental examples used to train the small parameter-specific calibration models described in Section 2.3. Second, they are withheld from the CatRange classifiers and from CatRange hyperparameter tuning and are evaluated later as a catalytic-residue anchor set. Thus, the anchor set is independent of CatRange classifier fitting but not independent of the upstream synthetic-label calibration procedure. To reduce similarity between these anchors and the CatRange training

distribution, anchor sequences were constrained to ≤60% global sequence identity relative to the corresponding CatRange training subsets.

2.3 Meta-learning calibration of mutation-effect labels

Kinetic effects in the WT–MD_{Ala} anchors were quantified on the same bin scale used by CatRange. For k_{cat}, a DROP label was assigned when the alanine mutant occupied a bin at least two levels below its cognate wild type; all other cases were labeled NO-DROP. For K_M, an INCREASE label was assigned when the mutant occupied a bin at least one level above the wild type; remaining cases were labeled NO-INCREASE. These directional labels were used only to calibrate synthetic mutation supervision and to summarize directional responses in the corresponding holdout analyses.

Separate binary calibration models were trained for k_{cat} DROP versus NO-DROP and K_M INCREASE versus NO-INCREASE. Input descriptors represented mutational severity, substrate chemistry, and enzyme–substrate scale. Mutational severity was quantified from the aggregate BLOSUM62 cost of alanine replacement at the annotated catalytic residues (*6*). RDKit-derived substrate descriptors included molecular weight, Crippen logP, ring count, hydrogen-bond donor count, and hydrogen-bond acceptor count. Additional descriptors included catalytic-residue density (annotated catalytic residues per sequence length), the substrate-to-enzyme molecular-weight ratio, and the corresponding wild-type kinetic bin.

Calibration used AutoTabPFNClassifier, a Transformer-based meta-learned classifier for tabular data (*7*). Given the small anchor collection, leave-one-out cross-validation maximized the available training information while retaining one held-out anchor at each iteration. StandardScaler was fit only to the training anchors within each iteration and then applied to the held-out case. The k_{cat} DROP classifier achieved 77.27% leave-one-out accuracy, and the K_M INCREASE classifier achieved 81.82%. Final calibration models were then refit on the full anchor collection, together with their fitted scalers, for application to the broader unlabeled catalytic-site alanine set.

2.4 Generation and labeling of synthetic catalytic-site alanine variants

Catalytic-site alanine variants were generated from UniProt-annotated catalytic residues to provide sequence-proximal perturbations during CatRange training. Each annotated catalytic residue was replaced by alanine; when a record contained multiple annotated catalytic residues, the annotated positions were substituted simultaneously. This procedure generated 5,278 synthetic variants. Alanine replacement removes side-chain chemistry that may contribute to acid–base catalysis, nucleophilic chemistry, metal or cofactor coordination, substrate positioning, or transition-state stabilization while preserving most of the global sequence background.

Synthetic variants were not assigned a blanket inactive label. The binary calibration output instead governed a parameter-specific relabeling policy. Variants classified as k_{cat} DROP were assigned a bin at least two levels below the cognate wild-type bin, bounded by the lowest available bin; NO-DROP variants were restricted to the wild-type or an adjacent bin. For K_M, variants classified as INCREASE were assigned at least one bin above the wild-type bin, whereas NO-INCREASE variants remained in the wild-type or a non-increasing bin. When several eligible bins satisfied the rule, assignment was sampled using a fixed random seed. The resulting synthetic examples were used as low-weight mutation-aware training instances rather than as replacements for experimental measurements.

3. Data Partitioning and Leakage-Controlled Evaluation Design

The evaluation design separates routine held-out prediction from mutation-focused distribution shift. Figure 4 in the main manuscript summarizes the mutation-aware validation layers; the construction of each dataset is specified below so that the source, withholding rule, and intended test remain explicit.

3.1 Standard cross-validation and independent holdout

Before cross-validation, 100 experimentally measured enzyme–substrate entries were sampled as an independent holdout using random_state = 42 and proportional sampling from wild-type and mutant-derived records to preserve the endogenous WT:MD composition. These entries were not used for model fitting, hyperparameter optimization, or preprocessing estimation. The remaining experimentally measured entries were partitioned by fivefold stratification using a composite label that encoded kinetic bin and biological source category (WT or mutant). Within each fold, the outer held-out portion was subdivided with the same composite stratification to produce approximate 80/10/10 train/validation/test proportions.

All data-dependent preprocessing was restricted to the training partition of each fold. Feature scaling parameters were estimated from training data and transferred unchanged to validation, test, holdout, OOD, and external data. SMOTE (*8*) was applied only to training data after bin assignment; validation and evaluation distributions were never oversampled.

3.2 Synthetic alanine-variant partitioning

The calibrated catalytic-site alanine pool was partitioned separately from the experimentally measured entries. Enzyme embeddings were first projected to two dimensions with PaCMAP (*9*), and affinity propagation (*10*) was applied to the projected representation to reduce overrepresentation of densely sampled regions. Clusters containing fewer than five members were assigned to a miscellaneous group to avoid unstable stratification. A composite stratification based on cluster membership and calibrated directional label then produced approximate 95%/2.5%/2.5% train/validation/test proportions for the synthetic pool.

3.3 Mutation-focused holdouts

Two targeted holdouts were defined in addition to the standard test partitions. The first contains 10 synthetic catalytic-site alanine variants withheld from CatRange training while their cognate wild-type enzyme backgrounds could remain represented among experimentally measured training entries. This sequence-proximal design asks whether the trained classifier responds to an unseen catalytic-site substitution rather than reproducing the wild-type bin. The ten enzyme–substrate backgrounds are listed in Table S2 and are evaluated in main-text Fig. 6 and Fig. S6.

The second targeted set is the 22 literature-derived WT–MD_{Ala} catalytic-residue anchor pairs described in Section 2.2. These pairs were used to calibrate the synthetic-labeling policy but were not used to fit the CatRange k_{cat} or K_M classifiers or to tune CatRange hyperparameters. Their ≤60% sequence-identity constraint is specific to the anchor analysis and should not be conflated with the cumulative homology thresholds used for standard test-set evaluation.

3.4 Homology-stratified evaluation subsets

For standard test-set homology analysis, each evaluated enzyme was compared with the corresponding fold-specific training set, and cumulative maximum sequence-identity thresholds of ≤40%, ≤60%, ≤80%, ≤90%, ≤99%, and ≤100% were used to summarize performance as progressively closer homologues entered the evaluation set. These thresholds define evaluation subsets rather than independently regenerated train–test partitions.

A separate mutation-focused subset was extracted from CatLog-27k test partitions by retaining experimentally measured mutant enzyme–substrate entries whose sequence identity to the corresponding training fold did not exceed 80%. This homology-stratified experimental mutant subset includes alanine and non-alanine substitutions across heterogeneous enzymes, substrates, and assay contexts. It therefore probes broader mutant generalization under sequence-distribution shift rather than specifically testing annotated catalytic-residue alanine substitution.

4. CatRange Model Development and Training

4.1 Enzyme sequence representations

Enzyme sequences were encoded with pretrained protein language models ESM-2 (esm2_t33_650M_UR50D) (*11*, *12*) and ESM-C (EvolutionaryScale/esmc-600m-2024-12) (*13*). Both models were used as frozen feature extractors without task-specific fine-tuning. Classification, terminal, and padding tokens were excluded before protein pooling, and final-layer residue embeddings across positions 1…L were mean-pooled to one descriptor per sequence. ESM-2 yielded a 1,280-dimensional representation and ESM-C a 1,152-dimensional representation. Mean pooling provides a scalable fixed-size descriptor but necessarily reduces residue-level positional resolution, including information localized to catalytic sites.

ESM-2 and ESM-C were compared under the same downstream CatRange partitions, substrate representation, classifier family, and metrics. Because ESM-C was comparable in routine held-out evaluation and modestly stronger on class-balanced mutation-focused evaluations (main-text Fig. 3A), ESM-C was used as the default enzyme representation for downstream analyses.

4.2 Substrate molecular representation

Substrates were represented from standardized isomeric SMILES using ChemBERTa (seyonec/PubChem10M_SMILES_BPE_450k) (*14*). Final hidden states were mean-pooled across the tokenized molecular sequence to generate 768-dimensional substrate descriptors. ChemBERTa special tokens were retained during pooling as part of the molecular string representation, whereas ESM pooling was restricted to amino-acid residue tokens. The resulting descriptors encode the chemical information present in the isomeric SMILES string, including stereochemical notation and recurring functional-group patterns.

4.3 Paired enzyme–substrate feature construction

For every kinetic record, the enzyme and substrate descriptors were concatenated so that the classifier received the sequence and assayed chemical context as one paired input. The default ESM-C–ChemBERTa representation contained 1,920 features; the ESM-2–ChemBERTa comparison contained 2,048. The concatenated representation does not explicitly encode a three-dimensional binding pose,

residue–substrate contacts, or active-site geometry. Instead, it provides a shared tabular feature space from which the classifier learns associations between sequence context, substrate identity, and kinetic-bin labels (main-text Fig. 2A).

4.4 Feature standardization

Protein and substrate embedding blocks were each standardized using means and standard deviations estimated from the training partition only. The same training-derived transformations were applied unchanged to validation, test, independent holdout, mutation-focused, and PafA inputs. Keeping the transformations fold-specific prevents information from the evaluation distributions from entering model preprocessing.

4.5 Gradient-boosted classifier training

CatRange uses Extreme Gradient Boosting implemented in XGBoost (*15*) as a multiclass classifier (main-text Fig. 2B). XGBoost was selected after a limited random-search screen of common tabular classifiers under the same cross-validation partitions. Separate classifiers were trained for k_{cat} and K_M. Successive decision trees were added to reduce the residual classification loss from earlier boosting rounds while regularization constrained tree complexity and leaf weights. The learning rate controlled the magnitude of successive updates; γ governed the minimum gain required for further node splitting, and L1/L2 penalties controlled leaf-weight complexity. This formulation provides nonlinear decision boundaries for the high-dimensional paired embeddings without introducing an explicit enzyme–substrate contact model.

4.6 Hyperparameter optimization and parameter sharing

Hyperparameters were optimized with Optuna (*16*) inside the fivefold training framework. Candidate configurations were fit only on each fold's training partition and evaluated on the corresponding validation partition. Validation accuracy was the optimization objective, while multiclass validation log-loss was monitored for early stopping with a patience of 10 boosting rounds. The selected configuration used 1,920 estimators, maximum tree depth 11, learning rate 0.0021, maximum delta step 0, L1 regularization $\alpha = 1.13$, and L2 regularization $\lambda = 14.803$.

The selected configuration was optimized for k_{cat} and then reused unchanged for K_M. This parameter-sharing choice keeps model capacity and optimization settings identical across the two kinetic tasks and avoids introducing a second task-specific tuning procedure; it also means that K_M performance was not independently hyperparameter-optimized. Population-level performance was summarized as mean ± SD across the five folds. For downstream inference requiring a single model, fold 5 was pre-specified as the representative inference model before final evaluation rather than selected post hoc on performance.

4.7 Class balancing, sample weighting, and hard-example reweighting

Kinetic bins are imbalanced, so SMOTE (*8*) was applied after binning within the training partition only, using k = 5 nearest neighbors and a fixed random seed. Validation, test, independent holdout, OOD, and PafA datasets retained their observed class distributions. Synthetic catalytic-site alanine variants were assigned a sample-weight factor of 0.001, whereas experimentally measured entries retained unit weight. This weighting allowed the synthetic examples to influence mutation-sensitive decision boundaries without dominating the experimentally measured kinetic distribution.

A second training stage emphasized difficult training examples. After the initial fit, training samples predicted outside the ±1-bin tolerance were identified within the training partition and assigned a 100-fold increase in sample weight before continued boosting from the previous model state. No validation, test, holdout, OOD, or PafA examples were used to identify hard examples or modify training weights. All stochastic steps used fixed random seeds where supported.

5. Evaluation Metrics and Comparative Benchmarking

5.1 Exact-bin, e-accuracy, and class-balanced metrics

Exact-bin accuracy is the fraction of predictions assigned to the same predefined numerical bin as the experimental value. Precision, recall, and F1 were computed for the multiclass problem and macro-averaged across classes so that underrepresented bins contributed to the summary. Multiclass Matthews correlation coefficient (MCC) was reported as a class-balanced measure based on the full confusion matrix, and area under the precision–recall curve (AUC-PR) was included because precision–recall summaries remain informative under class imbalance.

Error-tolerant accuracy (e-accuracy) measures a different resolution: a prediction is counted as e-accurate when it falls in the experimental bin or one immediately adjacent bin. It is therefore a ±1-bin tolerance metric, not a substitute for exact-bin recovery. The metric is reported alongside exact-bin accuracy and MCC because repeated measurements of identical sequence–substrate pairs can cross multiple log-scale boundaries before pooling and can span 6–8 orders of magnitude in extreme cases (Fig. S2). Adjacent-bin agreement is interpreted as tolerance-aware order-of-magnitude recovery, while exact-bin metrics retain the stricter multiclass requirement.

Confusion matrices were used to inspect per-bin error structure. t-SNE projections were used only as qualitative diagnostics of how true and predicted labels occupy the embedded feature space; they were not used as quantitative evidence of geometric class separation. All reported metrics were calculated in Python with scikit-learn.

5.2 Comparator retraining and common-bin evaluation

CatPred, DLKcat, UniKP, and EITLEM-Kinetics were retrained on the same fold assignments used for CatRange so that training, validation, and test entries were aligned across methods. Comparator models were trained only on experimentally labeled entries from the corresponding CatRange training partition. Published model settings were retained rather than independently retuning every comparator to CatLog-27k. Synthetic catalytic-site alanine variants were excluded from comparator retraining and from benchmark scoring when experimental labels were unavailable.

Because the comparator models primarily produce continuous values, each predicted k_{cat} value was mapped to the same CatRange bin definitions before scoring. Experimental values were mapped to the same bins, and predictions outside the defined numerical boundaries were assigned to the corresponding edge bin. This common label space permits direct comparison by exact-bin accuracy, e-accuracy, precision, recall, F1, and MCC under matched fold assignments and target definitions (main-text Fig. 3H).

For each fold, benchmark metrics were computed only on enzyme–substrate pair IDs for which a valid prediction was available from CatRange and all four retrained comparators. The resulting common

intersection was used identically for every model so that differences in reported metrics could not arise from model-specific sample availability. Continuous predictions were then converted to the CatRange k_{cat} bins before scoring. Table S4 reports five-fold mean ± SD metrics; the exploratory pooled error analysis described in the Results was calculated across the concatenated common test predictions from all five folds.

5.3 Homology-stratified benchmark evaluation

The comparator analysis was repeated over the cumulative fold-specific sequence-identity thresholds defined in Section 3.4. Test entries were held fixed across models within each threshold, and all outputs were scored in the shared bin space. This design evaluates how exact-bin and adjacent-bin performance changes as progressively closer homologues are admitted, rather than comparing independently constructed model-specific test sets (main-text Fig. 3I).

The comparator homology-stratified analysis in Fig. 3I used the prespecified Fold 5 inference model. At each cumulative sequence-identity threshold, the analysis was restricted to the common intersection of pair IDs with valid predictions from all models. Table S5 therefore reports both the number of truth records available at each threshold and the smaller common comparison set used for scoring.

6. Ablation Analyses

6.1 Paired enzyme–substrate representation ablation

To isolate the contribution of paired biochemical information, CatRange was retrained under three input configurations: the full concatenated enzyme–substrate representation, enzyme sequence only, and substrate only. The kinetic labels, folds, classifier family, class-balancing procedure, and evaluation metrics were unchanged across the three configurations. Separate ablations were fit for k_{cat} and K_M; training-only standardization and SMOTE followed the same leakage controls as the full model. Differences among these models therefore reflect the information removed from the input representation rather than a change in evaluation protocol.

6.2 Synthetic catalytic-site alanine supervision ablation

A no-synthetic CatRange ablation was trained with the same embeddings, folds, hyperparameters, class-balancing strategy, hard-example procedure, and evaluation metrics as full CatRange, but the calibrated catalytic-site alanine variants were removed from training. The comparison was evaluated on the catalytic-site alanine holdout and on the literature-derived WT–MD_{Ala} k_{cat} anchor set. For the anchor analysis, the experimental WT-to-mutant comparisons all corresponded to reduced mutant k_{cat}; the ablation therefore tests preservation of catalytic-drop behavior rather than general bidirectional gain/loss prediction.

Drop-sensitive summaries included expected-drop recall, predicted WT-to-mutant bin shift, mutant-bin recovery, joint WT–mutant exact-bin accuracy, and joint e-accuracy. Figure S8 shows the resulting WT prediction behavior, predicted mutant-versus-WT relationship, and per-pair trajectories for full CatRange and the no-synthetic ablation.

7. Mutation-Aware Validation

Mutation-aware validation was organized into hierarchical complementary tests that answer different questions (main-text Fig. 4). The homology-stratified experimental mutant subset tests broader mutation

generalization under sequence-distribution shift. The literature-derived WT–MD_{Ala} anchor set tests perturbation of UniProt-annotated catalytic residues while remaining excluded from CatRange classifier fitting. The catalytic-site alanine holdout tests unseen synthetic alanine substitutions in enzyme backgrounds that may otherwise be represented in training. These datasets are therefore reported separately rather than combined into a single mutation score.

7.1 Homology-stratified experimental mutant subset

Experimentally measured mutant enzyme–substrate pairs were extracted from the fold-specific CatLog-27k test partitions and retained when sequence identity to the corresponding training fold was ≤80%. The subset includes alanine and non-alanine substitutions and is heterogeneous in enzyme family, substrate, mutation type, and assay context. It is interpreted as a regime-level stress test of mutation generalization under sequence-distribution shift rather than a controlled comparison among specific substitution classes. Main-text Fig. 5A reports the k_{cat} analysis. The corresponding K_M analysis is shown in Fig. S5A.

7.2 Literature-derived WT–MD_{Ala} catalytic-residue anchor set

The 22 WT–MD_{Ala} anchor pairs provide experimentally measured examples of alanine substitution at annotated catalytic residues. The pairs were withheld from CatRange classifier training and hyperparameter optimization and constrained to ≤60% sequence identity relative to the corresponding CatRange training subsets. Their earlier use in Section 2 was limited to calibration of the synthetic-labeling policy. For CatRange evaluation, WT and mutant predictions were scored on the same bin definitions used during training. Main-text Fig. 5B reports the k_{cat} catalytic-residue analysis; Fig. S5B provides the corresponding K_M analysis, and Fig. S7 displays paired WT-to-mutant trajectories for both kinetic parameters.

7.3 Catalytic-site alanine holdout

The catalytic-site alanine holdout contains the 10 synthetic alanine substitutions listed in Table S2. Their mutant sequences were excluded from CatRange training, while the cognate wild-type enzyme backgrounds could remain represented among experimentally measured training entries. This controlled setting isolates whether the trained model changes its prediction when presented with an unseen catalytic-site perturbation in an otherwise familiar sequence background. Main-text Fig. 6 compares CatRange with retrained continuous predictors for WT and alanine-mutant behavior across k_{cat} and K_M.

Figure S6 provides a directional diagnostic for the same ten held-out cases by comparing the calibration-derived reference label with the WT-to-mutant shift predicted by the main CatRange model. This analysis is not an additional experimental ground truth: the DROP/NO-DROP and INCREASE/NO-INCREASE labels originate from the upstream calibration policy. In other words: *"calibration-derived reference labels"*. It therefore tests whether CatRange reproduces the intended mutation-aware supervision on unseen alanine variants, while main-text Fig. 6 provides the parameter-level comparison.

8. PafA Few-Shot Mutational Landscape

8.1 Dataset and few-shot partitioning

External few-shot evaluation used the alkaline phosphatase PafA mutational dataset from *Elizabethkingia meningoseptica* reported by Markin et al. (*17*), with carboxy 4-methylumbelliferyl phosphate ester (cMUP) as substrate. The dataset comprises 1,018 assayed sequences—1,016 single-point variants, one wild-type enzyme, and one double mutant—all within the same protein scaffold; the analysis therefore tests held-out mutation prediction within a densely sampled mutational landscape rather than generalization across enzyme families. The final partition comprised 398 unique training variants, 310 validation variants, and 310 test variants. Training-set construction was activity-informed using the bin labels, with sampling used to increase representation across distinct activity regions. Three prespecified records—the wild-type enzyme, T79S, and the double mutant N100A/R164A—were explicitly retained in training to provide the reference scaffold and selected catalytic perturbations. After all unique variants represented in training were removed, the remaining variants were divided equally and randomly between validation and test sets using a fixed seed (random_state = 42), without further stratification; thus, the held-out class distributions were not constrained to match that of the training set. The partitioning was not based on sequence identity. While the N100A/R164A double mutant provided training exposure to this position, all isolated single-mutant records for R164 substitutions—including the highlighted R164A and R164G test variants—were explicitly excluded from the training partition, with R164V assigned to the validation set.

8.2 Evaluation metrics and baseline interpretation

PafA predictions were evaluated by exact-bin accuracy, e-accuracy, and continuous-scale midpoint agreement. For the midpoint analysis, each predicted kinetic bin was represented by its geometric midpoint, which is equivalent to the midpoint in $\log_{10}$ space. For a bin with finite lower and upper bounds L_b and U_b, the representative value was $m_b = \sqrt{L_b U_b} = 10^{[\log_{10}(L_b)+\log_{10}(U_b)]/2}$. A prediction was counted as within one order of magnitude of the experimental value (y_{exp}) when $|\log_{10}(m_b/y_{\text{exp}})| \leq 1$. This continuous-scale criterion provides a physical performance metric distinct from e-accuracy, which compares discrete bin indices.

Moreover, the held-out k_{cat} test set contains 310 variants. True bin 5 is the most frequent class, with 185 of 310 entries (59.7%); this prevalence defines the exact-bin accuracy of a naive majority-class predictor that always assigns bin 5. The largest individual cell in the row-normalized confusion matrix contains 96 of 310 observations (31.0%) and should not be interpreted as class prevalence. Main-text Fig. 7B therefore reports the full test confusion matrix and e-accuracy together, rather than using a confusion-matrix cell occupancy as a baseline estimate.

8.3 Residue-wise kinetic context

To place highlighted catalytic variants within the complete PafA landscape, experimental apparent k_{cat} and K_M values and CatRange-predicted ranges were mapped by residue position. The 310 held-out test variants were randomly partitioned across the entire sequence (Section 8.1) and capture extensive spatial and chemical diversity, distributed across a 525-residue sequence span—from Q21 near the N-terminus to R546 at the C-terminus. Importantly, the evaluation extends beyond traditional alanine scanning by introducing substantial chemical diversity: 19 different types of wild-type amino acids

(alanine, arginine, asparagine, aspartic acid, glutamic acid, glutamine, glycine, histidine, isoleucine, leucine, lysine, methionine, phenylalanine, proline, serine, threonine, tryptophan, tyrosine, and valine) are mutated into three distinct target residues—alanine (A), glycine (G), and valine (V). Because these sampled variants are distributed globally across the sequence footprint, this mapping demonstrates how the model assesses diverse substitutions spanning varied structural microenvironments along a single, fixed scaffold. The residue-wise view emphasizes that the withheld R164A and R164G substitutions are evaluated in the dense context of hundreds of background variants, rather than as isolate, cherry-picked examples. Figure S9 provides this complementary positional view for both parameters, visually contrasting the model's localized mechanistic sensitivity at the active site with its structural boundaries when evaluating severe loss-of-function variants elsewhere in the sequence.

8.4 Post hoc structural-context analysis of failure cases

Structural information was not used for CatRange training, hyperparameter optimization, or model selection. After prediction, variants showing severe measured k_{cat} decreases that were not recovered by the model were inspected on the PafA structure (PDB ID 5TJ3) to identify whether local packing or contact density offered a plausible context for the error. Contact density was calculated as the number of neighboring residues within a 6Å threshold.

R164 has 13 neighboring residues within 6Å and lies in the densely connected active-site environment highlighted in main-text Fig. 7A. Residues 79 and 486 each have 18 neighbors under the same criterion (Fig. S10). These positions occur in locally contact-rich environments, consistent with the possibility that residue-level structural effects not captured by mean-pooled sequence embeddings contribute to the observed prediction errors. In addition, the H486V example illustrates the limitation of the pooled sequence representation: CatRange placed the mutant k_{cat} within approximately one order of magnitude of the wild-type regime even though the experimental mutation produced a substantially larger activity loss. This post hoc analysis identifies a class of residue-specific structural effects that can be difficult to resolve from mean-pooled sequence and substrate embeddings alone; it is interpretive and does not alter the reported CatRange predictions.

9. Code, Data, and Computational Resources

Computations were performed on the University of Nebraska-Lincoln SWAN HCC cluster and the Iowa State University NOVA high-performance computing system. The primary SWAN configuration used dual Intel Xeon Gold 6248R CPUs (48 total cores; up to 16 used for the reported workflows), 187 GiB RAM, and an NVIDIA Tesla V100S-PCIE-32GB GPU. The environment used CUDA 12.4 with NVIDIA driver 550.127.05. NOVA contributed to curation, embedding generation, and model training using dual 18-core Intel Xeon Gold 6140 CPUs (36 total cores), 192 GiB RAM, and dual NVIDIA Tesla V100 32-GB GPUs with CUDA 11.8. The implementation used Python 3.12.5. Principal software components included XGBoost 2.1.1 for gradient-boosted classification, PyTorch 2.4.0 for tensor and embedding workflows, imbalanced-learn 0.12.3 for SMOTE, and scikit-learn 1.5.1 for preprocessing and evaluation. A random seed of 42 was used across stochastic procedures where supported, including partitioning, SMOTE, and sampling steps.

CatRange code and notebooks are available at https://github.com/ssbio/CatRange. An executable Google Colab notebook is also available at https://colab.research.google.com/github/ssbio/CatRange/blob/main/CatRange_Inference_Interface.ipynb to facilitate

model use. The inference interface utilizes CLEAN as a preliminary filter to determine if a submitted protein sequence is an enzyme. If a sequence is classified as a non-enzyme, it is retained in the output dataset, but its downstream CatRange kinetics predictions are marked as skipped. A GUI version of CatRange is now hosted on OpenKinetics and available from https://predictor.openkinetics.org/. The public CatLog-27k release (https://chowdhurylab.github.io/tools/catlog-latest.html) preserves curated record-level measurements and provenance prior to the pooled model-ready representation.

Supplementary Figures

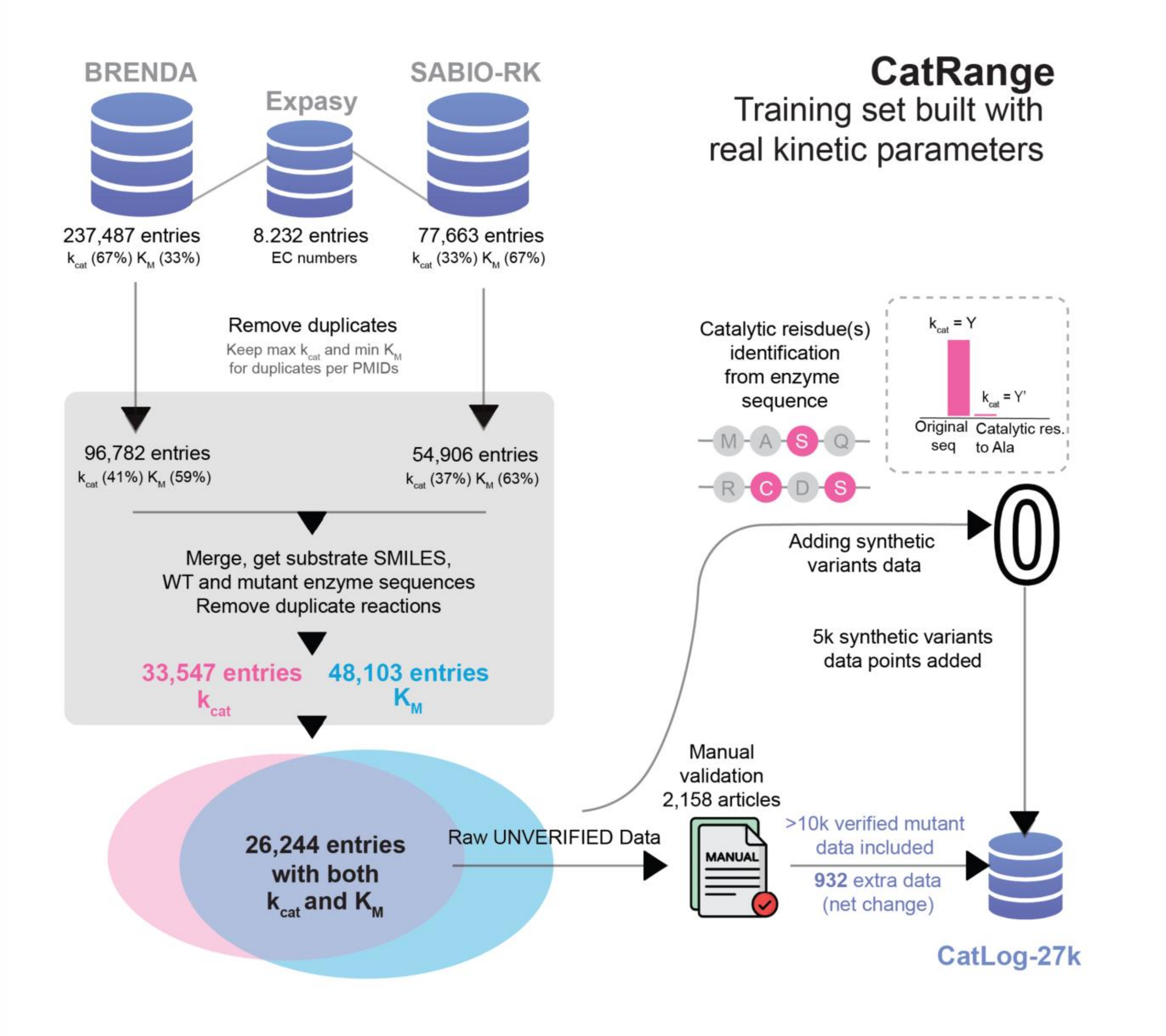

Fig. S1. Data collection and curation workflow used to construct CatLog-27k. Raw kinetic records were assembled from BRENDA and SABIO-RK and connected to EC annotations, standardized substrate representations, and enzyme sequences. The left branch summarizes the upstream database filtering and merging steps that produced 26,244 raw, unverified entries with both k_{cat} and K_M. Manual review of 2,158 source articles then corrected, removed, or added records; the 1,072 additions and 140 removals listed in Table S1 produce a net increase of 932 records, yielding 27,176 curated record-level entries. The right branch depicts generation of catalytic-site alanine variants for CatRange training. The synthetic variants were not assigned a universal zero-activity label; their kinetic labels were calibrated as described in Section 2.

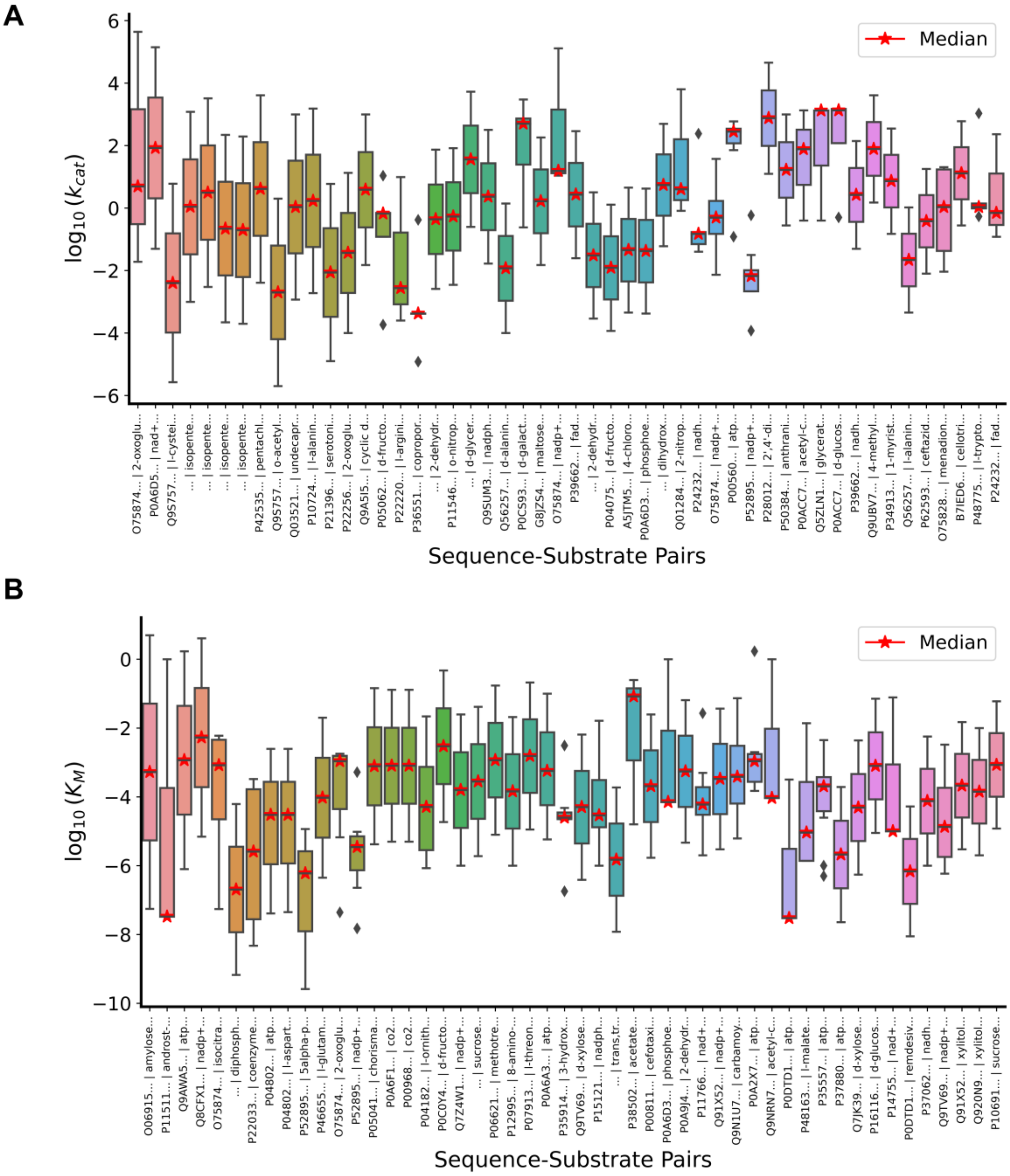

Fig. S2. Experimental variation among repeated measurements for the 50 sequence–substrate pairs with the largest reported spread. Boxplots summarize all available measurements for each identical sequence–isomeric-SMILES pair for (A) $\boldsymbol{k}_{\mathbf{cat}}$ and (B) $\boldsymbol{K_M}$; red stars mark the median. Several repeated pairs span 6–8 orders of magnitude across studies. The distribution motivates retention of record-level provenance in CatLog-27k and use of median pooling for the model-ready representation.

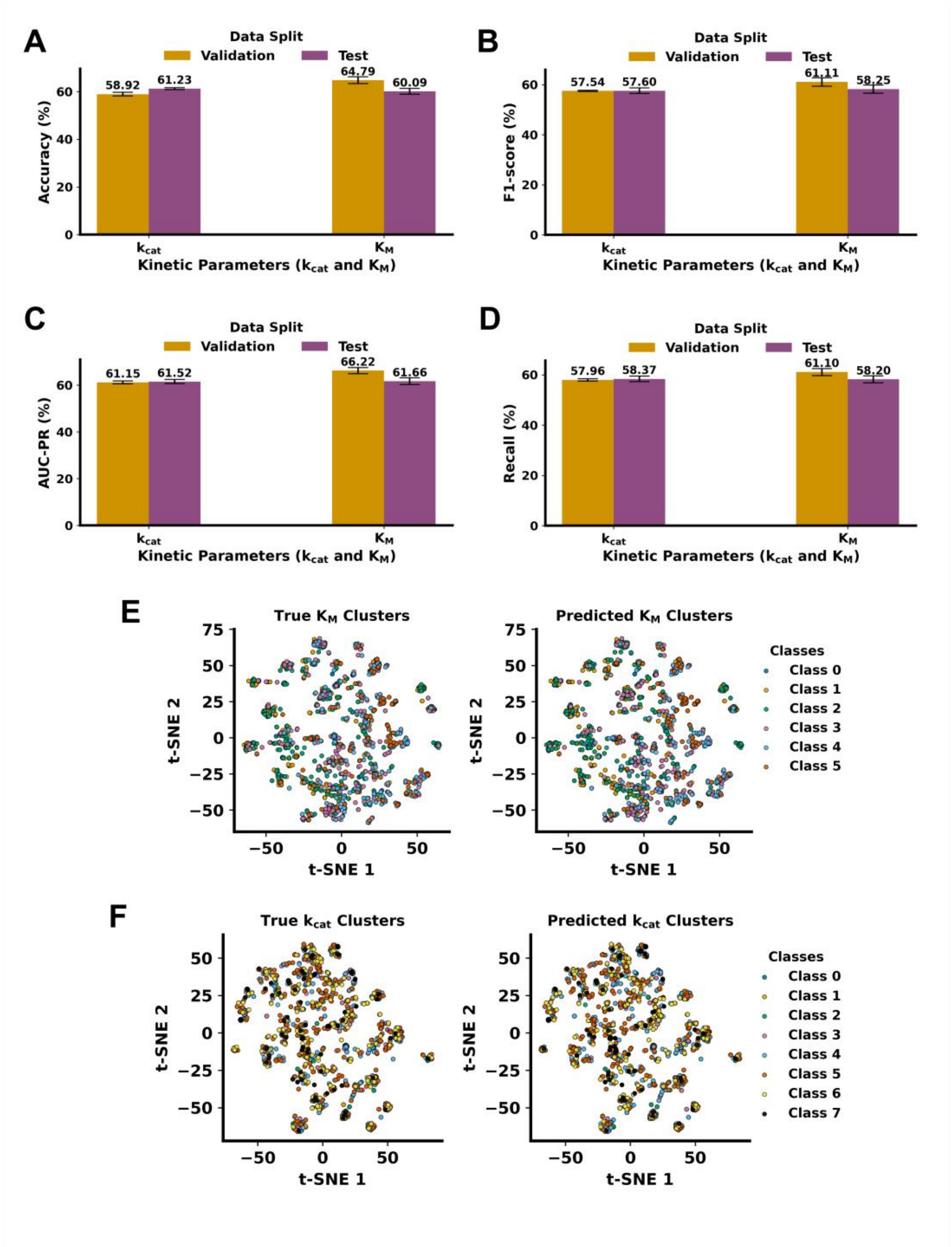

Fig. S3. Standard exact-bin performance metrics and qualitative class-structure diagnostics for CatRange. (A–D) Validation and test performance for exact-bin accuracy, F1, AUC-PR, and recall for k_{cat} and K_M; bars summarize the five cross-validation folds with variability shown by the plotted error bars. (E) t-SNE projections for true and predicted K_M classes and (F) corresponding projections for k_{cat}. The t-SNE panels provide a qualitative visualization of label organization in the embedded feature space and are interpreted together with the quantitative metrics rather than as an independent clustering test.

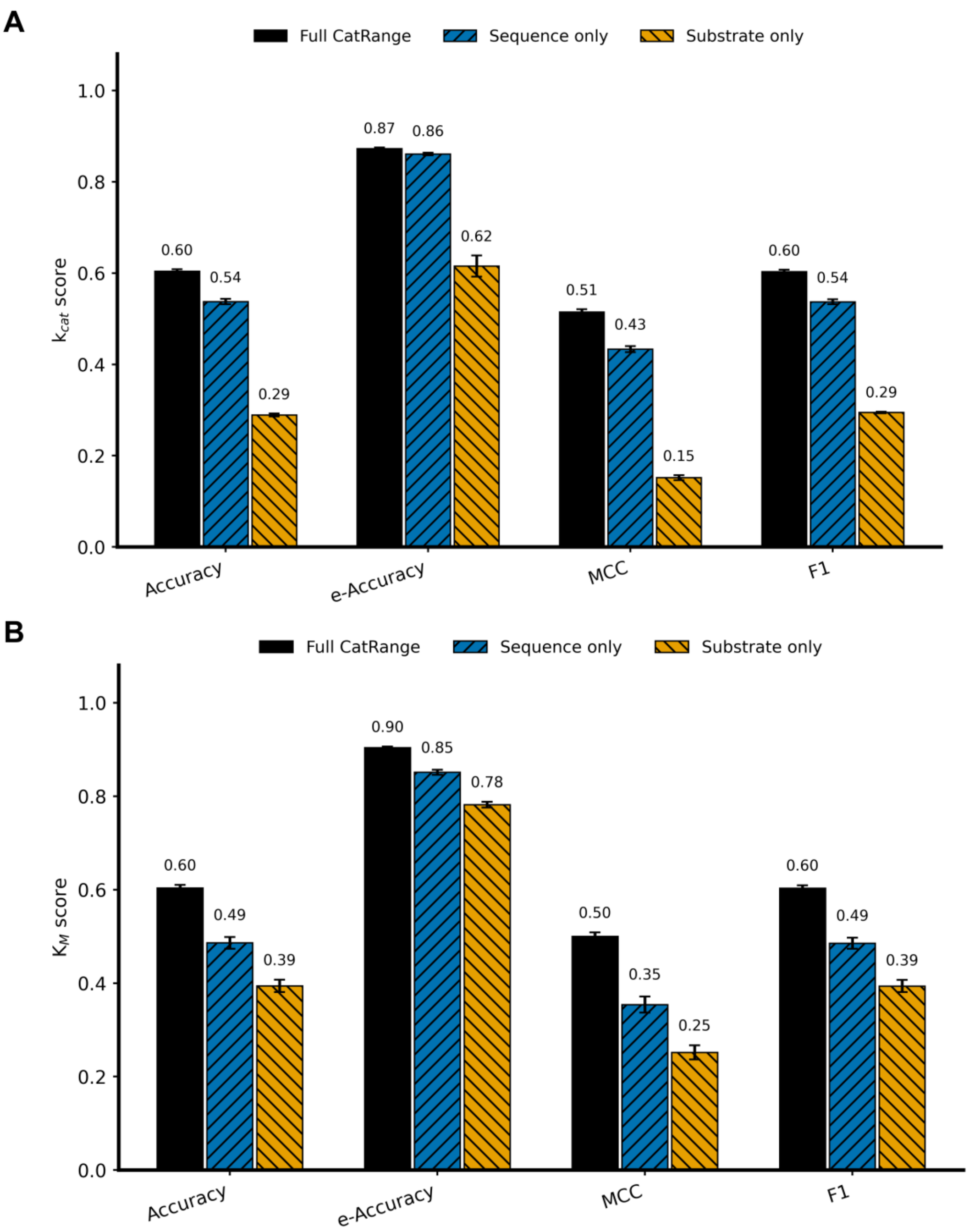

Fig. S4. Paired-representation ablation for CatRange. (A) k_{cat} and (B) K_M performance for the full CatRange representation, enzyme-sequence-only input, and substrate-only input. Exact-bin accuracy, e-accuracy, MCC, and F1 are shown under matched training and evaluation settings. The full paired representation provides the strongest overall exact-bin and class-balanced performance, while the relative sequence-only and substrate-only effects differ between k_{cat} and K_M.

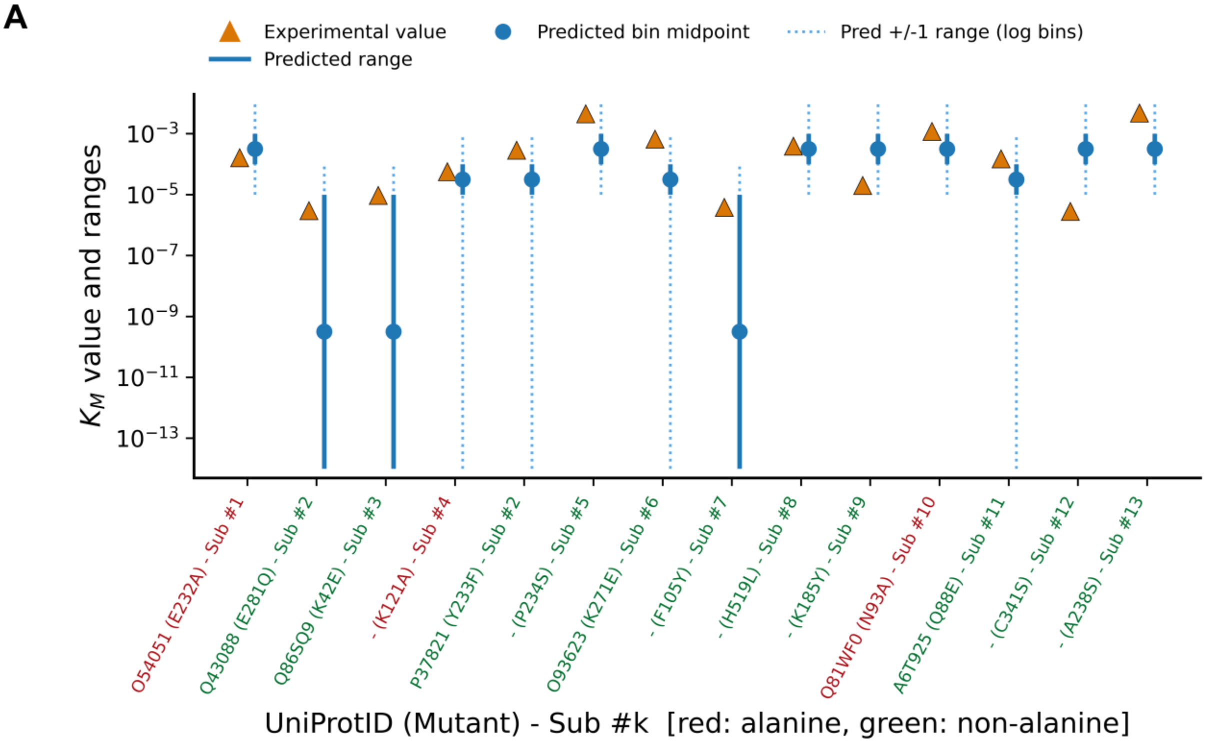

Substrate keys

Sub #1 = nad+
Sub #2 = s-adenosyl-l-methionine
Sub #3 = trans,trans-farnesyl diphosphate
Sub #4 = (2s,3r)-3-hydroxybutane-1,2,3-tricarboxylate
Sub #5 = udp-galactose
Sub #6 = atp
Sub #7 = 3'-azido-3'-deoxythymidine monophosphate
Sub #8 = bradykinin
Sub #9 = gastrin 17
Sub #10 = n-carbamoyl-l-aspartate
Sub #11 = 5-hydroxy-2-oxo-4-ureido-2,5-dihydro-1h-imidazole-5-carboxylate
Sub #12 = fe2+
Sub #13 = d-glyceraldehyde 3-phosphate

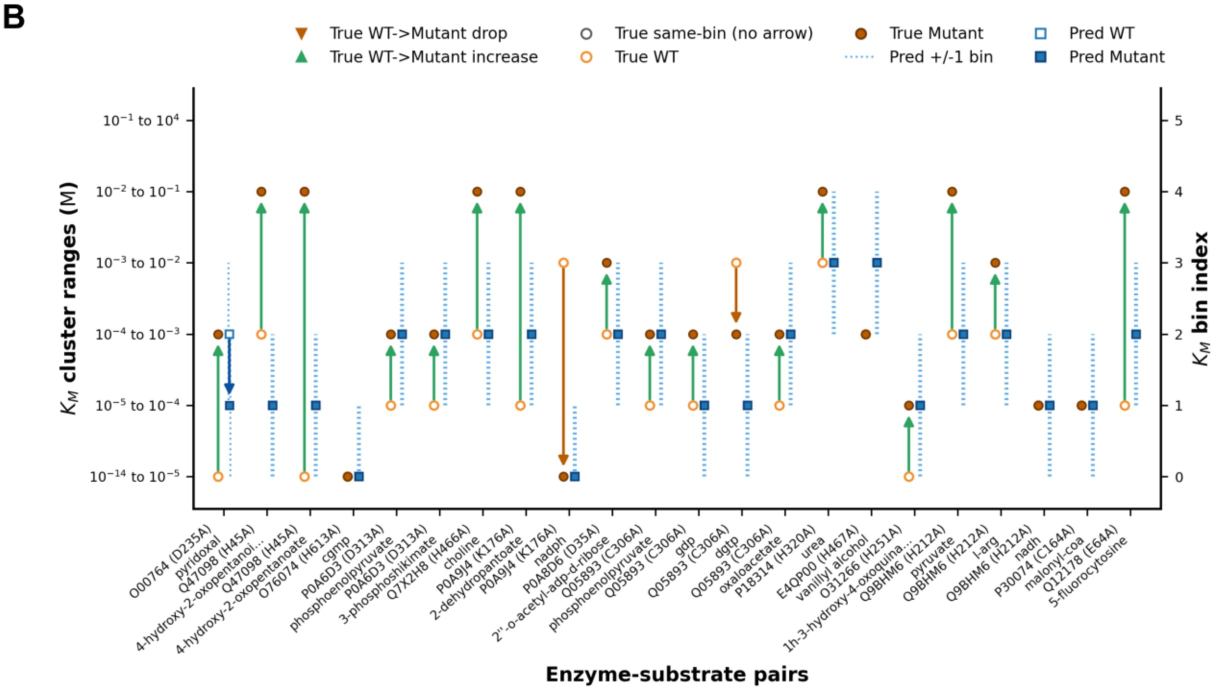

Fig. S5. Complementary K_M mutation-aware evaluations corresponding to the two tests shown for k_{cat} in Fig. 5. (A) Homology-stratified experimental mutant subset: measured mutant K_M values are compared with CatRange-predicted bin midpoints and ranges for the ≤80% identity mutant subset; red labels denote alanine substitutions and green labels non-alanine substitutions. (B) Literature-derived WT–MD_{Ala} catalytic-residue anchor set: experimental WT-to-mutant K_M movements are shown together with CatRange WT and mutant bin predictions and the ±1-bin prediction range. The two panels represent distinct validation datasets and should not be interpreted as one pooled mutant set.

A

	Main: DROP	Main: NO-DROP	Row total
Meta: DROP	TP 7 (70%)	FN 2 (20%)	9 (90%)
Meta: NO-DROP	FP 0 (0%)	TN 1 (10%)	1 (10%)

Precision=1.00, Recall=0.78, F1=0.87, MCC=0.51, tau=2 bins

Case	UniProt ID	WT True	WT Pred	MD Pred	Δ bin	Shift	Meta Label	Predicted (WT-MD)	Match
1	P17516	2	2	0	2	↓ 2	DROP	DROP	YES
2	Q9P2T1	3	3	3	0	0	NO-DROP	NO-DROP	YES
3	Q6FFQ0	2	5	1	4	↓ 4	DROP	DROP	YES
4	P9WNX5	4	4	1	3	↓ 3	DROP	DROP	YES
5	P37213	5	5	5	0	0	DROP	NO-DROP	NO
6	P00924	5	5	2	3	↓ 3	DROP	DROP	YES
7	O81413	6	6	6	0	0	DROP	NO-DROP	NO
8	Q91WR5	2	2	0	2	↓ 2	DROP	DROP	YES
9	Q2MHD9	2	3	0	3	↓ 3	DROP	DROP	YES
10	P0A6L2	6	6	4	2	↓ 2	DROP	DROP	YES

B

	Main: INCREASE	Main: NO-INCREASE	Row total
Meta: INCREASE	TP 2 (20%)	FN 3 (30%)	5 (50%)
Meta: NO-INCREASE	FP 0 (0%)	TN 5 (50%)	5 (50%)

Precision=1.00, Recall=0.40, F1=0.57, MCC=0.50, tau=-1 bins

Case	UniProt ID	WT True	WT Pred	MD Pred	Δ bin	Shift	Meta Label	Predicted (WT-MD)	Match
1	P17516	0	0	0	0	0	NO-INCREASE	NO-INCREASE	YES
2	Q9P2T1	1	1	1	0	0	INCREASE	NO-INCREASE	NO
3	Q6FFQ0	2	2	5	-3	↑ 3	INCREASE	INCREASE	YES
4	P9WNX5	1	1	1	0	0	NO-INCREASE	NO-INCREASE	YES
5	P37213	1	1	1	0	0	INCREASE	NO-INCREASE	NO
6	P00924	1	1	1	0	0	INCREASE	NO-INCREASE	NO
7	O81413	3	3	3	0	0	NO-INCREASE	NO-INCREASE	YES
8	Q91WR5	0	0	0	0	0	NO-INCREASE	NO-INCREASE	YES
9	Q2MHD9	1	1	1	0	0	NO-INCREASE	NO-INCREASE	YES
10	P0A6L2	2	2	5	-3	↑ 3	INCREASE	INCREASE	YES

Fig. S6. Directional shift classification for unseen catalytic-site variants (supporting Main Fig. 6). Directional agreement between the expected mutation-effect labels (derived from the "Meta"-learner calibration) and the explicit predictions made by the "Main" CatRange framework for the 10 strictly withheld synthetic catalytic-site alanine variants (Table S2). The calibration-derived labels serve as supposed functional shift reference for this synthetic holdout—distinct from direct experimental mutant measurements—as detailed in *SI Appendix*, Section 2.3. (A) k_{cat} DROP evaluation using a two-bin decrease threshold. The confusion summary reports TP = 7, FN = 2, FP = 0, and TN = 1 (precision 1.00, recall 0.78, F1 = 0.87, MCC = 0.51); the case table lists the WT true bin, WT prediction, alanine-mutant prediction, predicted bin shift, calibration label, CatRange directional prediction, and match status. (B) K_M INCREASE evaluation using the study's one-bin directional threshold. The confusion summary reports TP = 2, FN = 3, FP = 0, and TN = 5 (precision 1.00, recall 0.40, F1 = 0.57, MCC = 0.50), followed by the same case-level fields. The calibration-derived labels are used here as the reference supervision for the synthetic holdout and are distinct from direct experimental mutant measurements.

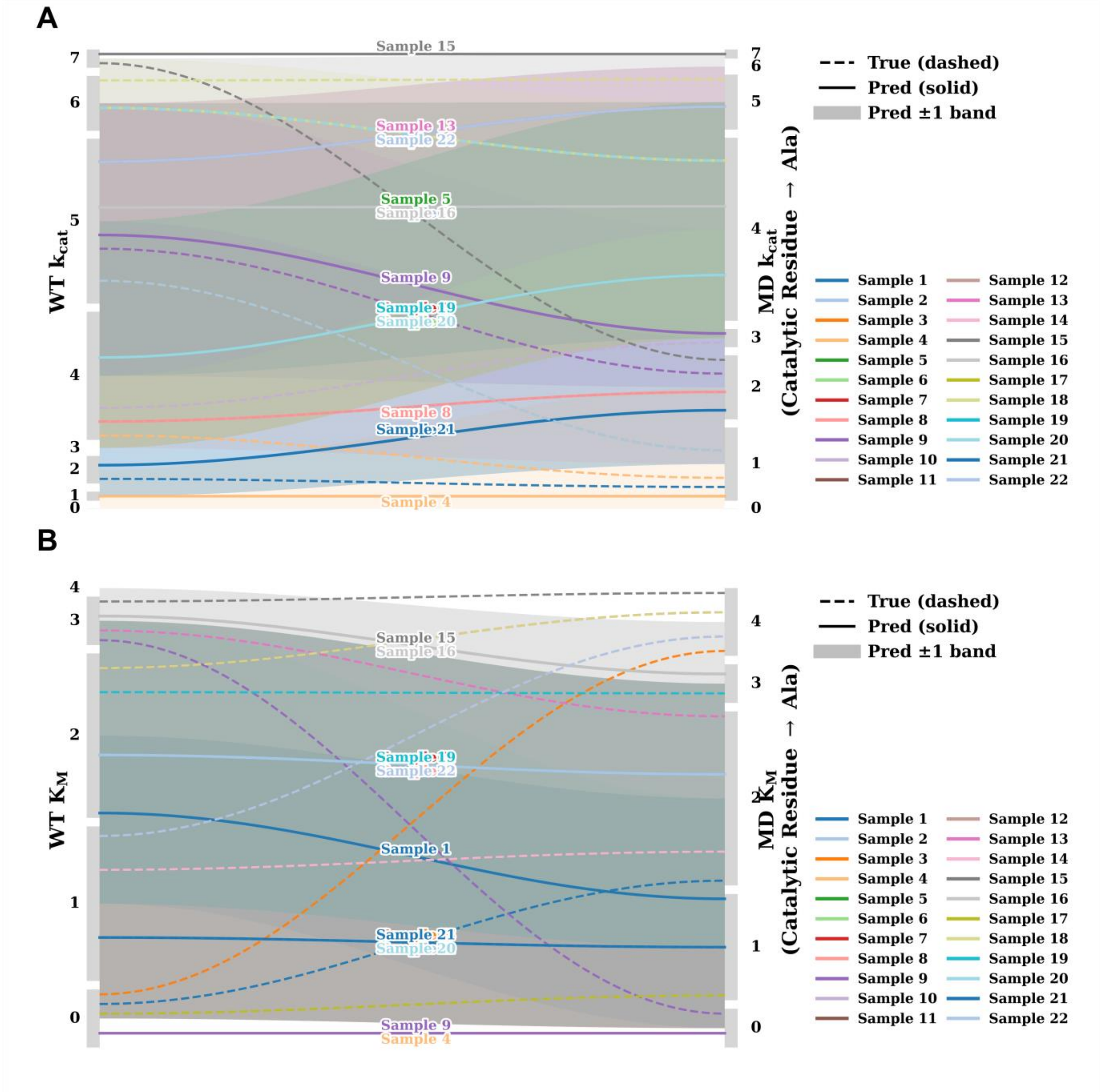

Fig. S7. Longitudinal visualization of the 22 literature-derived WT–MD$_{Ala}$ catalytic-residue anchor pairs withheld from CatRange classifier training. (A) WT-to-mutant trajectories for k_{cat} and (B) corresponding trajectories for K_M. Dashed curves denote experimental WT-to-mutant behavior, solid curves CatRange predictions, and shaded regions the predicted ±1-bin neighborhood. Each color identifies one enzyme–substrate pair. This view complements the bin-level anchor analyses by showing the direction and magnitude of paired WT-to-mutant movement across the full anchor collection.

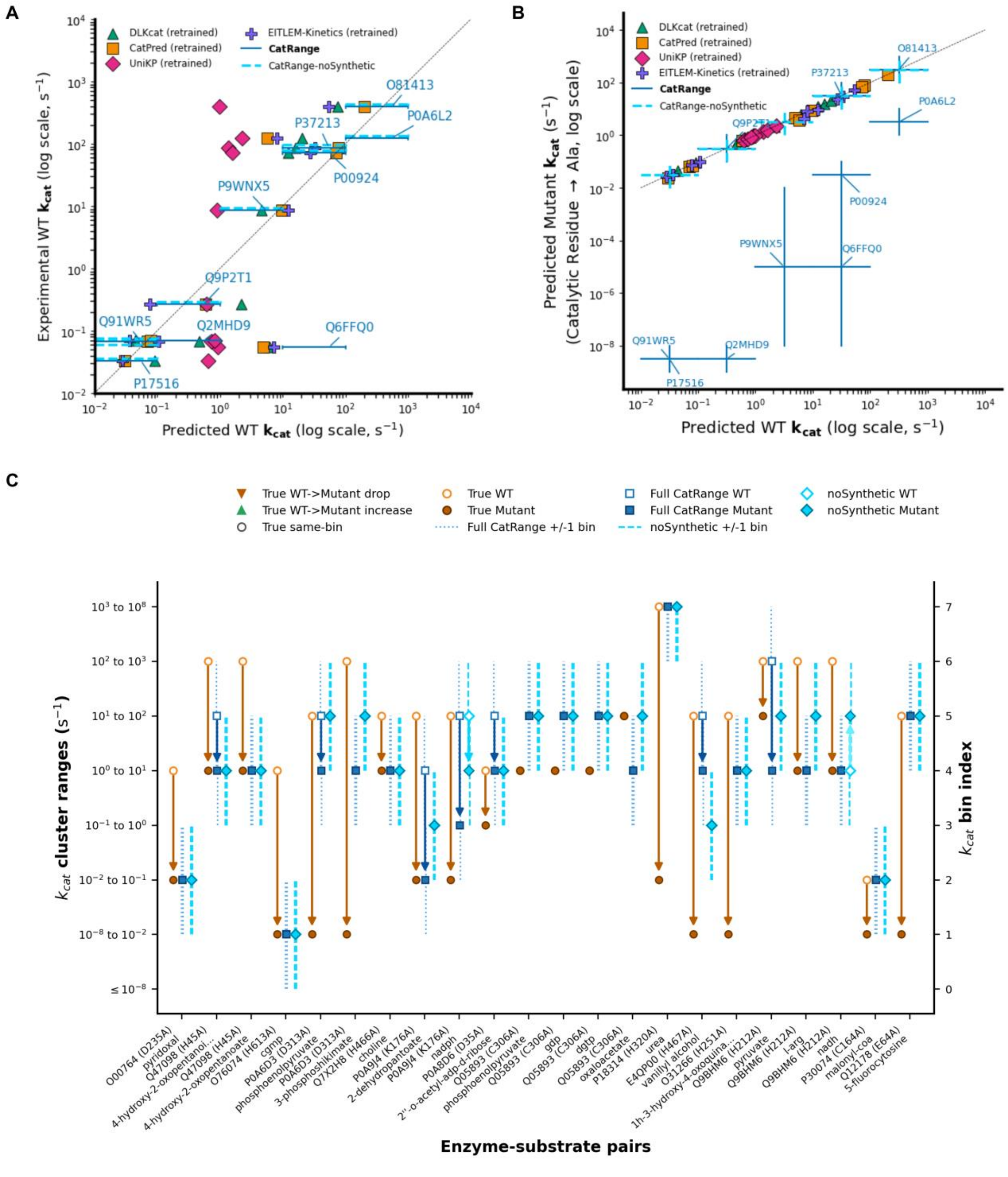

A
DLKcat (retrained)
CatPred (retrained)
UniKP (retrained)
EITLEM-Kinetics (retrained)
CatRange
CatRange-noSynthetic
O81413
P0A6L2
P37213
P00924
P9WNX5
Q9P2T1
Q91WR5
Q2MHD9
Q6FFQ0
P17516
Experimental WT kcat (log scale, s−1)
Predicted WT kcat (log scale, s−1)
B
DLKcat (retrained)
CatPred (retrained)
UniKP (retrained)
EITLEM-Kinetics (retrained)
CatRange
CatRange-noSynthetic
O81413
P37213
P0A6L2
Q9P2T1
P00924
P9WNX5
Q6FFQ0
Q91WR5
Q2MHD9
P17516
Predicted Mutant kcat (s−1)
(Catalytic Residue → Ala, log scale)
Predicted WT kcat (log scale, s−1)
C
True WT->Mutant drop
True WT->Mutant increase
True same-bin
True WT
True Mutant
Full CatRange +/-1 bin
Full CatRange WT
Full CatRange Mutant
noSynthetic +/-1 bin
noSynthetic WT
noSynthetic Mutant
kcat cluster ranges (s−1)
kcat bin index
Enzyme-substrate pairs

Fig. S8. Ablation of synthetic catalytic-site variants eliminates mutation sensitivity. Evaluation of the CatRange-noSynthetic ablation model, which was trained on CatLog-27k without the 5,278 mechanistically guided catalytic-site alanine variants for k_{cat} prediction. (A) Experimental versus predicted wild-type (WT) k_{cat} parameters for the synthetic-negative holdout set (n=10, supporting Main Fig. 6). Both the full CatRange model (solid blue crosshairs) and the ablated noSynthetic model (dashed cyan crosshairs) successfully recover the WT kinetic regimes. (B) Predicted mutant versus predicted WT k_{cat} for the same holdout set. While the full model correctly shifts below the $\boldsymbol{y} = \boldsymbol{x}$ identity line to capture functional impairment, the ablated model completely collapses onto the diagonal, exhibiting the same severe mutation blindness as the continuous benchmark models. (C) Kinetic regime-shift predictions for the 22 strictly held-out WT–MD_{Ala} catalytic-residue anchors (supporting Main Fig. 5B). The full model (dark blue squares) successfully tracks the experimental WT-to-mutant directional drops (orange). In contrast, the ablated noSynthetic model (cyan diamonds) predominantly defaults to assigning identical bins to both WT and mutant sequences. Together, these results confirm that calibrated synthetic negatives are strictly required to teach the framework to recognize localized active-site perturbations.

A

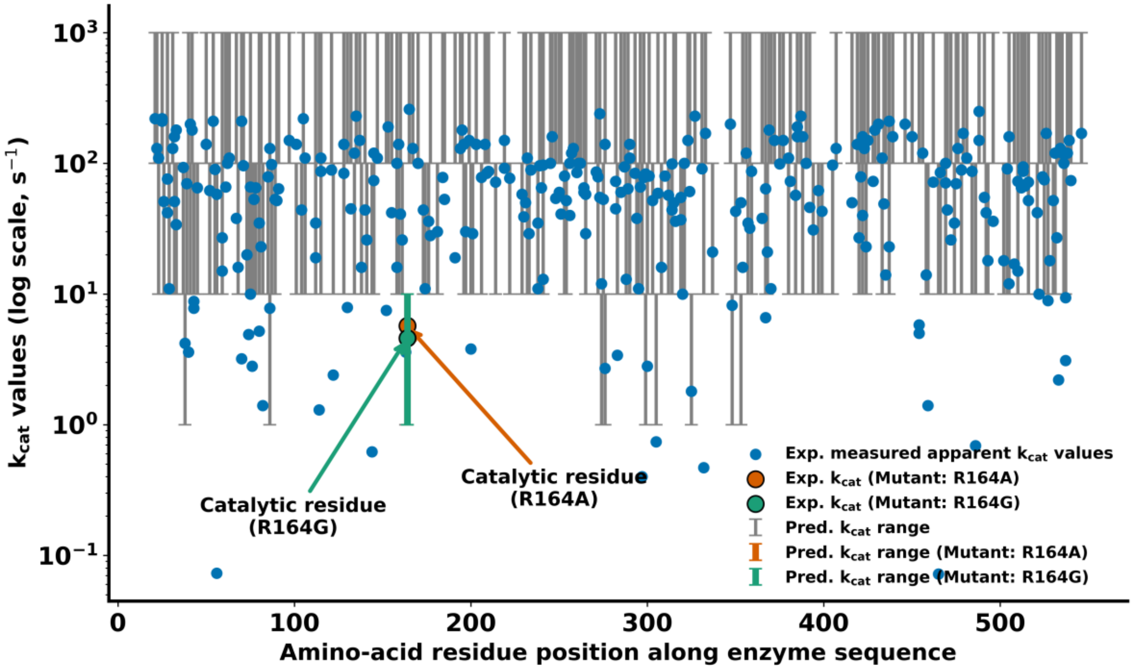
10^3
10^2
10^1
10^0
10^{-1}
k_{cat} values (log scale, s^{-1})
0
100
200
300
400
500
Amino-acid residue position along enzyme sequence
Catalytic residue (R164G)
Catalytic residue (R164A)
Exp. measured apparent k_{cat} values
Exp. k_{cat} (Mutant: R164A)
Exp. k_{cat} (Mutant: R164G)
Pred. k_{cat} range
Pred. k_{cat} range (Mutant: R164A)
Pred. k_{cat} range (Mutant: R164G)

B

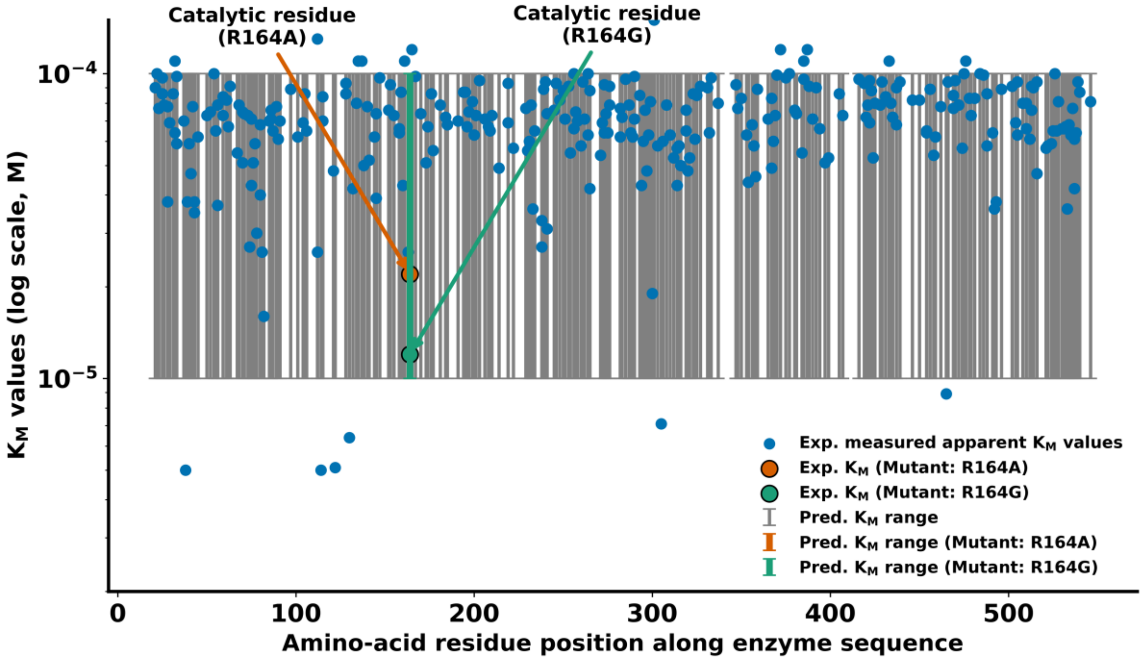
10^{-4}
10^{-5}
K_M values (log scale, M)
0
100
200
300
400
500
Amino-acid residue position along enzyme sequence
Catalytic residue (R164A)
Catalytic residue (R164G)
Exp. measured apparent K_M values
Exp. K_M (Mutant: R164A)
Exp. K_M (Mutant: R164G)
Pred. K_M range
Pred. K_M range (Mutant: R164A)
Pred. K_M range (Mutant: R164G)

Fig. S9. Positional mapping of predicted kinetic regimes across the PafA mutational landscape (supporting Main Fig. 7). (A and B) Experimental measurements (blue markers) versus CatRange-predicted kinetic ranges (grey bars) mapped across the amino-acid residue positions of the enzyme sequence for apparent k_{cat} (A) and K_M (B). This evaluation strictly visualizes the 310 held-out test variants. The vertical bars denote the numerical span of the discrete $\log_{10}$-scaled kinetic bin assigned by the model, rather than statistical variance. The catalytic active-site substitutions R164A (orange) and R164G (green) are highlighted as held-out single-mutant examples whose measured values fall within the predicted ranges. The broader residue-wise landscape also reveals severe low- k_{cat} variants for which CatRange remains closer to central activity ranges. Particularly, predicted ranges correctly shifted downward from the baseline wild-type values (WT k_{cat}≈$1.20\times10^{2}\text{s}^{-1}$; WT K_M≈4.5×10^{-5}M), to encompass the true measurements. In contrast, Panel A visually exposes the framework's structural boundaries: for severe loss-of-function variants scattered across the sequence (e.g., experimental $\boldsymbol{k_{cat}} \leq \mathbf{10^0}\text{s}^{-1}$), the predicted ranges frequently fail to capture the full magnitude of impairment, defaulting instead to conservative, wild-type-like central regimes. This distinct failure mode confirms that while sequence embeddings detect active-site chemical perturbations, they are insufficient to resolve catastrophic, non-local structural collapse.

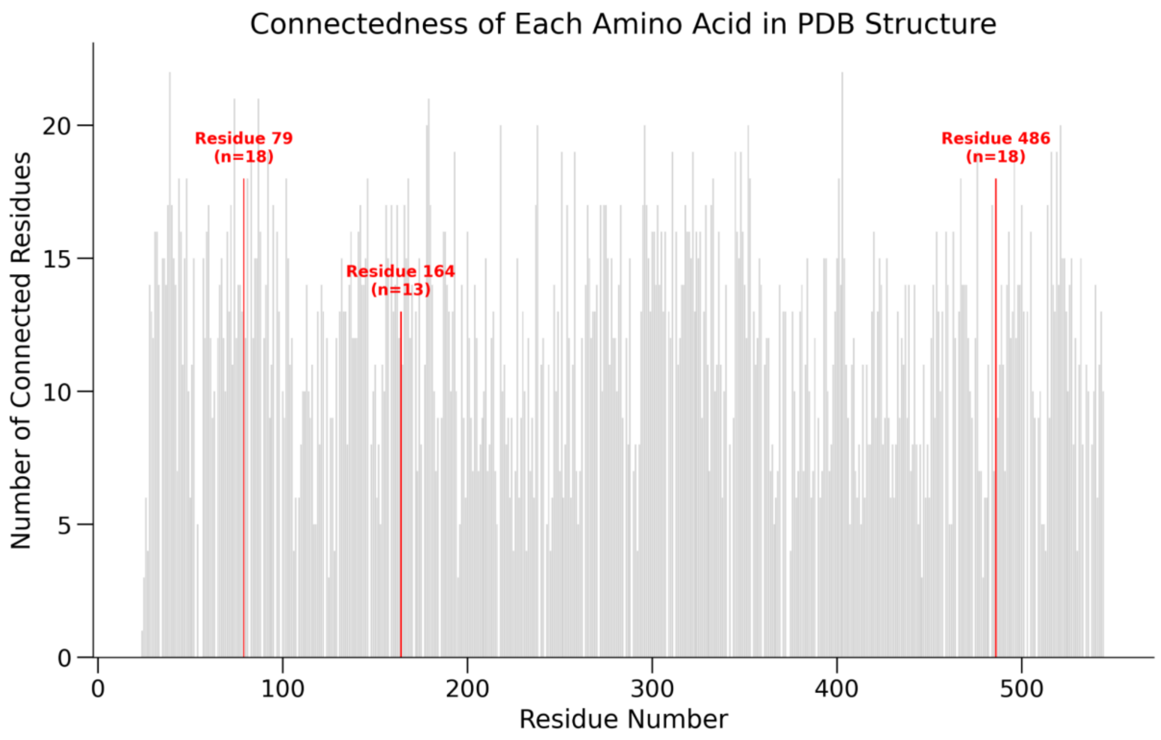

Fig. S10. Residue contact density in the PafA structure (PDB ID 5TJ3). Gray bars show the number of residues within 6 Å of each amino-acid position. Highlighted positions are residue 79 (18 neighbors), residue 164 (13 neighbors), and residue 486 (18 neighbors). The contact-density analysis was performed post hoc to contextualize mutation-associated prediction failures and was not used as an input feature for CatRange.

Supplementary Tables

Table S1. Resolution and additions in the CatLog-27k mutant dataset. This table details the curation process, highlighting resolutions and removals across parameters (K_M and k_{cat}) substrates, and mutations. 1804 discrepancies were resolved, with 1,072 new entries added, refining the dataset to 11,175 mutant entries for predictive modeling.

Parameter	corrected	removed
K_M	788	10
k_{cat}	618	10
substrates	240	8
mutations	18	21
duplicates	-	91
additional data	1072	-

Table S2. Independent in-distribution hold-out set of 10 catalytic-site alanine variants. Summary of wild-type (WT) enzymes whose corresponding alanine-substituted mutants used for in-distribution hold-out evaluation. These alanine mutants (MD_{Ala}) were excluded from CatRange training, while their cognate WT enzyme backgrounds could be represented in the training distribution, providing a controlled evaluation of CatRange's mutation sensitivity to unseen catalytic-site perturbations.

Case #	UniProt ID	Substrate Name	Catalytic Residues	k_{cat} [s^{-1}]	K_M [mM]	Organism	Pathway
1	P17516	3alpha-hydroxy-5beta-pregnane-20-one	Y55	$3.3x10^{-2}$	$1.6x10^{-3}$	*Homo sapiens*	steroid metabolism
2	Q9P2T1	gmp	C186, T188	$2.7x10^{-1}$	$1.7x10^{-2}$	*Homo sapiens*	purine metabolism
3	Q6FFQ0	succinate semialdehyde	E264, C301	$5.5x10^{-2}$	$4.5x10^{-1}$	*Acinetobacter baylyi*	carbohydrate acid metabolism
4	P9WNX5	nadp+	C130, H256	$8.5x10^{0}$	$6.5x10^{-2}$	*Mycobacterium tuberculosis*	amino-acid biosynthesis
5	P37213	diphosphate	H454, C839	$8.7x10^{1}$	$6.8x10^{-3}$	*Entamoeba histolytica*	
6	P00924	mn2+	E212, K346	$7.2x10^{1}$	$1.9x10^{-2}$	*Saccharomyces cerevisiae*	carbohydrate degradation/ glycolysis
7	O81413	lipoamide	H479	$3.9x10^{2}$	$3.4x10^{0}$	*Glycine max*	
8	Q91WR5	3beta-hydroxyandrost-5-en-17-one	Y55	$6.8x10^{-2}$	$4.0x10^{-4}$	*Mus musculus*	steroid metabolism
9	Q2MHD9	5alpha-androstane-3alpha,17beta-diol	Y55	$7.0x10^{-2}$	$1.7x10^{-2}$	*Rattus norvegicus*	
10	P0A6L2	l-aspartate 4-semialdehyde	Y133, K161	$1.2x10^{2}$	$1.5x10^{-1}$	*Escherichia coli*	amino-acid biosynthesis

Table S3 (separate file). Curated set of 22 literature-derived, experimentally characterized wild-type (WT)–alanine-mutated (MD$_{Ala}$) enzyme-substrate pairs at UniProt-annotated catalytic residues, used to anchor calibration of the mechanistically guided catalytic-site alanine variants. These entries were held out under a ≤60% sequence-identity cutoff relative to the CatRange training set. They span a range of mutation-associated kinetic responses—including substantial k_{cat} decreases, typically ≥2 orders-of-magnitude reductions in k_{cat} as well as K_M shifts, ≥1 order-of-magnitude increases in K_M and cases remaining within the same kinetic regime—providing benchmarks for calibrating synthetic negatives and for training the binary meta-label classifier.

Table S4 (separate file). Bin-compatible k_{cat} benchmark source data for main-text Fig. 3H. Fivefold mean ± SD values for CatRange, CatPred, DLKcat, UniKP, and EITLEM-Kinetics across exact-bin accuracy, e-accuracy, precision, recall, F1, and MCC after conversion of all model outputs to the common CatRange bin (kinetic-regime) space. Values are fold-level mean ± SD.

Table S5 (separate file). Homology-stratified k_{cat} benchmark source data for main-text Fig. 3I. The table reports CatRange and retrained-comparator metrics across cumulative sequence-identity thresholds of ≤40%, ≤60%, ≤80%, ≤90%, ≤99%, and ≤100%, together with the number of evaluated entries at each threshold.

SI References

1. A. Chang, L. Jeske, S. Ulbrich, J. Hofmann, J. Koblitz, I. Schomburg, M. Neumann-Schaal, D. Jahn, D. Schomburg, BRENDA, the ELIXIR core data resource in 2021: new developments and updates. *Nucleic Acids Res.* **49**, D498–D508 (2021).

2. D. Dudaš, U. Wittig, M. Rey, A. Weidemann, W. Müller, Improved insights into the SABIO-RK database via visualization. *Database* **2023**, 1–9 (2023).

3. S. Kim, J. Chen, T. Cheng, A. Gindulyte, J. He, S. He, Q. Li, B. A. Shoemaker, P. A. Thiessen, B. Yu, L. Zaslavsky, J. Zhang, E. E. Bolton, PubChem 2023 update. *Nucleic Acids Res.* **51**, D1373–D1380 (2023).

4. A. Bateman, M. J. Martin, S. Orchard, M. Magrane, S. Ahmad, E. Alpi, E. H. Bowler-Barnett, R. Britto, H. Bye-A-Jee, A. Cukura, P. Denny, T. Dogan, T. G. Ebenezer, J. Fan, P. Garmiri, L. J. da Costa Gonzales, E. Hatton-Ellis, A. Hussein, A. Ignatchenko, G. Insana, R. Ishtiaq, V. Joshi, D. Jyothi, S. Kandasaamy, A. Lock, A. Luciani, M. Lugaric, J. Luo, Y. Lussi, A. MacDougall, F. Madeira, M. Mahmoudy, A. Mishra, K. Moulang, A. Nightingale, S. Pundir, G. Qi, S. Raj, P. Raposo, D. L. Rice, R. Saidi, R. Santos, E. Speretta, J. Stephenson, P. Totoo, E. Turner, N. Tyagi, P. Vasudev, K. Warner, X. Watkins, R. Zaru, H. Zellner, A. J. Bridge, L. Aimo, G. Argoud-Puy, A. H. Auchincloss, K. B. Axelsen, P. Bansal, D. Baratin, T. M. Batista Neto, M. C. Blatter, J. T. Bolleman, E. Boutet, L. Breuza, B. C. Gil, C. Casals-Casas, K. C. Echioukh, E. Coudert, B. Cuche, E. de Castro, A. Estreicher, M. L. Famiglietti, M. Feuermann, E. Gasteiger, P. Gaudet, S. Gehant, V. Gerritsen, A. Gos, N. Gruaz, C. Hulo, N. Hyka-Nouspikel, F. Jungo, A. Kerhornou, P. Le Mercier, D. Lieberherr, P. Masson, A. Morgat, V. Muthukrishnan, S. Paesano, I. Pedruzzi, S. Pilbout, L. Pourcel, S. Poux, M. Pozzato, M. Pruess, N. Redaschi, C. Rivoire, C. J. A. Sigrist, K. Sonesson, S. Sundaram, C. H. Wu, C. N. Arighi, L. Arminski, C. Chen, Y. Chen, H. Huang, K. Laiho, P. McGarvey, D. A. Natale, K. Ross, C. R. Vinayaka, Q. Wang, Y. Wang, J. Zhang, UniProt: the Universal Protein Knowledgebase in 2023. *Nucleic Acids Res.* **51**, D523–D531 (2023).

5. A. Bar-Even, E. Noor, Y. Savir, W. Liebermeister, D. Davidi, D. S. Tawfik, R. Milo, The moderately efficient enzyme: Evolutionary and physicochemical trends shaping enzyme parameters. *Biochemistry* **50**, 4402–4410 (2011).

6. S. Henikoff, J. G. Henikoff, Amino acid substitution matrices from protein blocks. *Proc. Natl. Acad. Sci. U. S. A.* **89**, 10915–10919 (1992).

7. N. Hollmann, S. Müller, L. Purucker, A. Krishnakumar, M. Körfer, S. Bin Hoo, R. T. Schirrmeister, F. Hutter, Accurate predictions on small data with a tabular foundation model. *Nature 2025 637:8045* **637**, 319–326 (2025).

8. N. V. Chawla, K. W. Bowyer, L. O. Hall, W. P. Kegelmeyer, SMOTE: Synthetic Minority Over-sampling Technique. *Journal of Artificial Intelligence Research* **16**, 321–357 (2002).

9. Y. Wang, H. Huang, C. Rudin, Y. Shaposhnik, Understanding How Dimension Reduction Tools Work: An Empirical Approach to Deciphering t-SNE, UMAP, TriMap, and PaCMAP for Data Visualization. *Journal of Machine Learning Research* **22**, 1–73 (2021).

10. B. J. Frey, D. Dueck, Clustering by Passing Messages Between Data Points. *Science (1979).*, doi: 10.1126/science.1136800 (2007).

11. A. Rives, J. Meier, T. Sercu, S. Goyal, Z. Lin, J. Liu, D. Guo, M. Ott, C. L. Zitnick, J. Ma, R. Fergus, Biological structure and function emerge from scaling unsupervised learning to 250 million protein sequences. *Proc. Natl. Acad. Sci. U. S. A.* **118**, e2016239118 (2021).

12. Z. Lin, H. Akin, R. Rao, B. Hie, Z. Zhu, W. Lu, N. Smetanin, R. Verkuil, O. Kabeli, Y. Shmueli, A. dos Santos Costa, M. Fazel-Zarandi, T. Sercu, S. Candido, A. Rives, Evolutionary-scale prediction of atomic-level protein structure with a language model. *Science (1979).* **379**, 1123–1130 (2023).

13. ESM Team, Evolutionary Scale · ESM Cambrian: Revealing the mysteries of proteins with unsupervised learning, *EvolutionaryScale Website* (2024). https://www.evolutionaryscale.ai/blog/esm-cambrian.

14. S. Chithrananda, G. Grand, B. R. Deepchem, ChemBERTa: Large-Scale Self-Supervised Pretraining for Molecular Property Prediction. (2020).

15. T. Chen, C. Guestrin, XGBoost: A scalable tree boosting system. *Proceedings of the ACM SIGKDD International Conference on Knowledge Discovery and Data Mining* **13-17-August-2016**, 785–794 (2016).

16. T. Akiba, S. Sano, T. Yanase, T. Ohta, M. Koyama, Optuna: A Next-generation Hyperparameter Optimization Framework. *Proceedings of the ACM SIGKDD International Conference on Knowledge Discovery and Data Mining*, 2623–2631 (2019).

17. C. J. Markin, D. A. Mokhtari, F. Sunden, M. J. Appel, E. Akiva, S. A. Longwell, C. Sabatti, D. Herschlag, P. M. Fordyce, Revealing enzyme functional architecture via high-throughput microfluidic enzyme kinetics. *Science (1979).* **373** (2021).
